# Telerehabilitation Trends in Australian Physiotherapy and an Exploration of Factors That Influence Use After COVID-19 Restrictions: Qualitative Content Analysis

**DOI:** 10.2196/81008

**Published:** 2026-01-27

**Authors:** Megan H Ross, Joshua Simmich, Belinda J Lawford, Kim L Bennell, Rana S Hinman, Trevor Russell

**Affiliations:** 1 RECOVER Injury Research Centre, Faculty of Health, Medicine and Behavioural Sciences, The University of Queensland, Surgical, Treatment and Rehabilitation Service (STARS), 296 Herston Rd, Herston, Queensland, 4006, Australia, +61 7 3365 5560; 2 Centre for Health, Exercise and Sports Medicine, Department of Physiotherapy, School of Health Sciences, Faculty of Medicine, Dentistry & Health Sciences, The University of Melbourne, Melbourne, Australia

**Keywords:** telehealth, COVID-19, videoconferencing, survey, physiotherapy, qualitative

## Abstract

**Background:**

Telerehabilitation is a safe and effective means of delivering physiotherapy services, but implementation in clinical practice has not been widespread.

**Objective:**

This study aimed to explore the shifts in telerehabilitation use throughout the COVID-19 pandemic and the key factors that influenced telerehabilitation caseload after restrictions were eased.

**Methods:**

Between September and November 2023, physiotherapists practicing in Australian private practice, hospital outpatient, or community settings completed an online survey. Data were collected regarding participants’ use of telerehabilitation before, during, and after the COVID-19 pandemic restrictions to in-person physiotherapy. Qualitative content analysis of open-text questions was performed to garner more nuanced information about the use of telerehabilitation in clinical practice, and quantitative data were analyzed descriptively.

**Results:**

The proportion of participants using telerehabilitation rose from 30% (44/148) before the pandemic to 94% (138/147) when restrictions to in-person physiotherapy were in place. Although 82% (118/144) of the sample continued to deliver telerehabilitation after COVID-19 restrictions were eased, telerehabilitation accounted for only 14% of the total caseload. Exploratory analyses suggest that despite increased confidence, satisfaction, and perceptions about the effectiveness of telerehabilitation, reduced patient demand, physiotherapists’ perceptions about patient preference for in-person consultations, and the perception that in-person physiotherapy is easier continue to influence the use of telerehabilitation in the post-COVID era.

**Conclusions:**

Despite increased uptake during the pandemic, telerehabilitation caseload after restrictions were eased was low. Physiotherapists’ perceptions about telerehabilitation in clinical practice remain a substantial barrier to sustained adoption.

## Introduction

### Background

Evidence suggests that telerehabilitation is a safe, feasible, and effective means of delivering physiotherapy care that is at least as good as in-person physiotherapy in terms of patient outcomes [1,2]. Despite two decades of evidence supporting the effectiveness of telerehabilitation in the management of musculoskeletal [3], neurological [4], cardiorespiratory [5], and postsurgical rehabilitation [6], clinician acceptance and adoption have been low [7]. While telerehabilitation is a viable alternative to traditional in-person physiotherapy with the potential to overcome geographical barriers, improve access, and facilitate continuity of treatment, integration into routine physiotherapy practice before the COVID-19 pandemic remained limited [8].

Before the COVID-19 pandemic, physiotherapists were slow and reluctant to adopt telerehabilitation as a standard model of care [9,10]. Several barriers contributed to this limited uptake of telerehabilitation in physiotherapy practice, including limited acceptance of and low confidence in using telehealth technology, perceived limitations in conducting physical assessments remotely, and reduced capacity to deliver hands-on interventions that are central to traditional physiotherapy practice [8,11,12]. Additional challenges included perceptions that telerehabilitation was less effective for certain clinical presentations and concerns about developing rapport and patient engagement [11,12].

The COVID-19 pandemic brought unprecedented challenges to health care systems worldwide, including the delivery of physiotherapy services. In response to the pandemic-related restrictions on in-person consultations, many physiotherapy practices turned to telerehabilitation [13] as an alternative method to continue providing necessary care [14]. Although physiotherapy was recognized as an essential health care service in Australia during the COVID-19 pandemic, practice was still notably restricted. Stringent infection control measures, such as mandatory use of personal protective equipment, rigorous patient screening, physical distancing requirements, density limits within clinical spaces, and group size limitations on group therapy sessions [15], impacted the delivery of care across multiple clinical settings. During initial lockdowns, some states, such as Victoria, further restricted in-person physiotherapy services, permitting face-to-face consultations only for urgent cases [16]. Community and aged care physiotherapy faced further barriers, including restrictions on therapists attending multiple sites and outright bans on external providers entering residential facilities [17]. Consequently, physiotherapy practice during the pandemic was markedly disrupted, forcing providers to rapidly transition to providing telerehabilitation services to adhere to public health guidelines and ensure continuity of care.

With the rapid transition to telerehabilitation in response to the pandemic came changes in regulatory frameworks to fund telerehabilitation [18], position statements advocating for the use of telerehabilitation [19], and increased infrastructure and clinical training to support the integration of telerehabilitation into clinical care [20]. During this period, uptake of telerehabilitation increased substantially, reflecting the necessity to maintain continuity of care. Research conducted at the time suggested that physiotherapists intended to continue offering services via telerehabilitation after the easing of restrictions to in-person physiotherapy [20,21]. However, international evidence suggests that uptake and usage have generally decreased from the pandemic peak [22].

### Objectives

With the rapid transition to telerehabilitation in response to the pandemic came changes in regulatory frameworks to fund telerehabilitation [18], position statements advocating for the use of telerehabilitation [19], and increased infrastructure and clinical training to support the integration of telerehabilitation into clinical care [20]. During this period, uptake of telerehabilitation increased substantially, reflecting the necessity to maintain continuity of care. Research conducted at the time suggested that physiotherapists intended to continue offering services via telerehabilitation after the easing of restrictions to in-person physiotherapy [20,21]. However, international evidence suggests that uptake and usage have generally decreased from the pandemic peak [22].

The aim of this study was to investigate the use of telerehabilitation in Australian physiotherapy clinical practice throughout the COVID-19 pandemic, with a focus on telerehabilitation use after restrictions were eased.

The specific research questions for this study were as follows: (1) How did the use of telerehabilitation vary in physiotherapy clinical practice in Australia before, during, and after COVID-19 restrictions to in-person consultations? and (2) What are the key factors that influence physiotherapists’ telerehabilitation caseload in the postrestrictions period?

## Methods

### Design

A descriptive, cross-sectional survey was conducted online with physiotherapists currently practicing in Australia. The study was primarily quantitative, with a small qualitative component to supplement descriptive analyses.

### Ethical Considerations

The study was approved by The University of Queensland Human Research Ethics Committee (approval number: 2023/HE001802) and reported following the consensus-based CROSS (Checklist for Reporting of Survey Studies) [23]. Participants provided electronic informed consent after reviewing an information sheet and before completing the survey. Participants were entered into a draw for a AUD $1000 (US $667) gift voucher upon completion of the survey. Participants’ privacy and confidentiality were maintained by storing nonidentifiable survey data separately from contact details on the University of Queensland Research Data Management System.

### Participants

Participants were physiotherapists recruited from the community via online advertisements on social media (eg, Facebook, X, and LinkedIn), via targeted emails, and through Australian Physiotherapy Association member communications (eg, eComms). Physiotherapists were eligible to participate if they were registered with the Australian Health Practitioner Regulation Agency and currently practicing in an Australian private practice, hospital, or community setting. Participants who had not delivered telerehabilitation services were eligible to complete a short version of the questionnaire to explore reasons for not engaging with telerehabilitation and the circumstances that might influence uptake.

### Procedure

An online survey was designed to capture information that was relevant to stakeholders and ensure readability and credibility (Multimedia Appendix 1). The survey was developed by the authors using Bennell [20] as a guide and adapted to capture information relevant to the different phases of the COVID-19 pandemic restrictions. The 3 phases were “Prior to the pandemic restrictions,” “During the period of restrictions to in-person physiotherapy” (from the introduction of restrictions in 2020 to 2022), and “After restrictions were eased” (2022 onward). Questions were primarily multiple choice (checkbox questions), numerical rating scales (0‐10), and 5-point Likert scales. Respondents were asked to estimate their telerehabilitation caseload (individual video, group video, and telephone) for each phase using a sliding scale (0%‐100%). Free-text responses were sought for some questions to ascertain more nuanced and in-depth information about physiotherapists’ perceptions of using telerehabilitation in clinical practice.

The survey was administered via an online secure platform (Qualtrics, LLC) and hosted by The University of Queensland. Participants were first invited to complete the online consent form and screening and, if eligible, proceeded to the survey. Participants were asked to provide demographic information, details of clinical practice, and experience with telerehabilitation. The second primary section of the survey comprised questions pertaining to the use of telerehabilitation during each phase of the COVID-19 pandemic restrictions (before, during, and after). All data were collected between September 15 and November 8, 2023.

### Data Analysis

Data were exported from the online platform for analysis in R (version 4.3.3; R Core Team). Descriptive statistics, including frequencies (percentages) and means and standard deviations, were used to summarize the data. All responses (including partial responses) meeting eligibility criteria were included in analyses. When an “other” field was provided for additional response options, 2 researchers reviewed free-text responses and either aligned them with existing response options or designated them as unique responses that were added to the final list of response options. Any discrepancies in coding were resolved via discussion.

Responses to free-text questions were analyzed qualitatively using inductive content analysis in Microsoft Excel [24]. First, 2 researchers (MHR and JS) independently read the entire dataset, conducted open coding, and identified topics and initial patterns. The unit of analysis was meaning units, identified within individual responses. Codes were subsequently categorized and combined to form main categories or themes (abstraction), with both authors returning to the dataset to check that codes made sense in relation to the raw data. The 2 authors then met to compare and discuss their coding frameworks, and discrepancies were resolved through discussion. An audit trail was maintained to document coding decisions and category development. Themes with the highest number of individual data points were identified, reported, and described. To enhance trustworthiness, reflexivity was considered throughout the process, and attention was paid to credibility and transparency in coding and interpretation.

To explore which factors influenced physiotherapists’ use of telerehabilitation in the postpandemic restrictions period, the total proportion of videoconferencing telerehabilitation caseload (individual and group consultations) was examined. Specifically, this proportion was plotted against the following five key postpandemic variables: confidence, satisfaction, and perceived effectiveness of telerehabilitation; physiotherapists’ perception about how much patients like telerehabilitation; and how often patients are requesting it. Locally estimated scatterplot smoothing curves were fitted using the full span of the data (span=1). These smoothed trends, along with their corresponding 95% CIs, were used to visually explore apparent associations. No statistical correlation or regression analyses were performed on these trends.

## Results

### Sample Characteristics

A total of 222 physiotherapists responded to the survey, with 152 (68%) meeting eligibility criteria and providing sufficient data to be included in analyses (58/222, 26%, excluded for not being an Australian Health Practitioner Regulation Agency–registered physiotherapist currently practicing in an eligible setting [eg, private practice, hospital outpatient, or community] and 12/222, 5% not providing sufficient data to determine eligibility). Most participants (107/152, 70%) completed the survey in less than 20 minutes.

Respondents were primarily women (87/152, 57%); working in musculoskeletal (105/152, 69%) private practice (84/152, 55%) in Queensland (42/152, 28%), Victoria (42/152, 28%), or New South Wales (38/152, 25%); and held either a Bachelor’s (70/152, 46%) or Master’s (58/152, 38%) degree in physiotherapy. Physiotherapists primarily used Zoom (66/142, 47%), a telephone (59/142, 42%) or Microsoft Teams (47/142, 33%) to conduct telerehabilitation consultations. Only 40% (n=56) of respondents indicated that they had participated in telerehabilitation training. Additional participant characteristics are provided in Table 1.

**Table 1.** Participant characteristics (total N=152 unless otherwise specified).

Characteristic	Values, n (%)^a^
Gender	
Woman	87 (57)
Man	63 (41)
Prefer not to say	2 (1)
State or territory	
Queensland	42 (28)
Victoria	42 (28)
New South Wales	38 (25)
Western Australia	13 (9)
South Australia	10 (7)
Australian Capital Territory	6 (4)
Tasmania	1 (1)
Area of practice	
Private practice (primary care)	83 (55)
Public health outpatient center	45 (30)
Community health center	29 (19)
Private hospital	10 (7)
Other	20 (13)
Clinical focuses	
Musculoskeletal or orthopedic	105 (69)
Sports and exercise	43 (28)
Neurology	30 (20)
Gerontology	24 (16)
Pediatric	12 (8)
Other	35 (23)
Highest education	
Bachelor’s degree	70 (46)
Master’s by coursework	58 (38)
Masters by research	3 (2)
Postgraduate diploma	11 (7)
PhD	6 (4)
Other	4 (3)
Prior training in telehealth	
No	96 (63)
Yes, <6 mo ago	5 (3)
Yes, between 6 and 12 mo ago	6 (4)
Yes, between 12 mo and 2 y ago	17 (11)
Yes, between 2 and 3 y ago	17 (11)
Yes, longer than 3 y ago	11 (7)
Telerehabilitation software (recently used; n=142)	
Zoom	66 (47)
Telephone	59 (42)
Microsoft Teams	47 (33)
Physitrack	30 (21)
Other	89 (63)

a Percentages may not sum to 100% because respondents could select multiple options.

### Shifts in Telerehabilitation Use Through the Phases of the COVID-19 Pandemic

**Figure 1.**
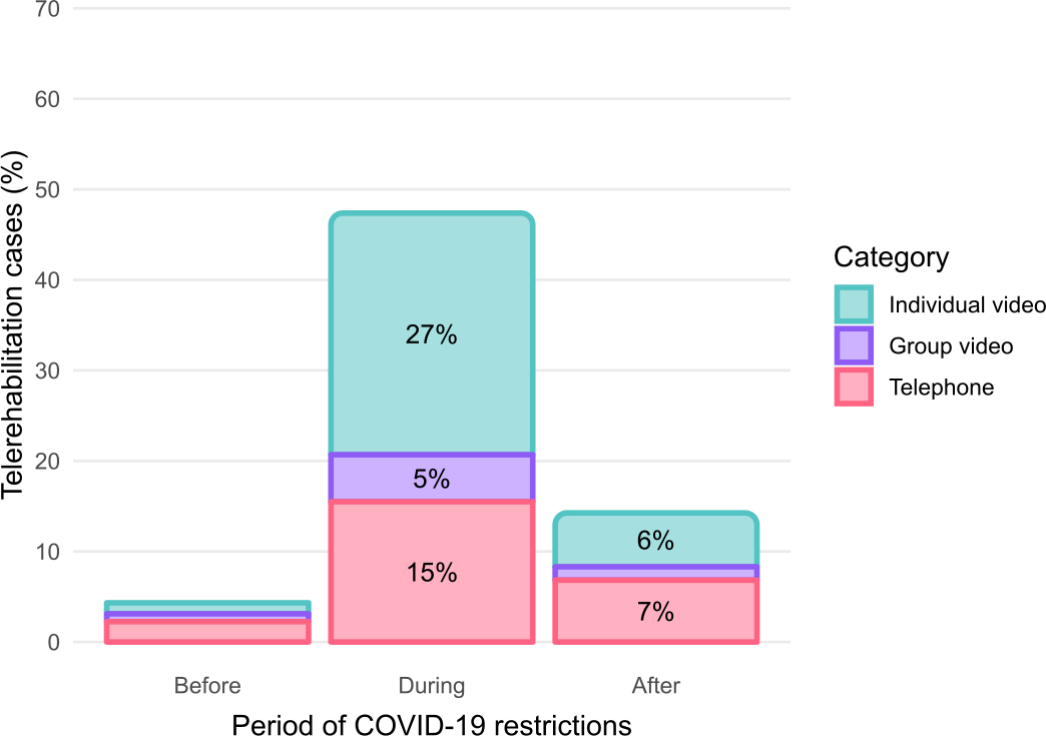
Shift in estimated telerehabilitation caseload before, during, and after the COVID-19 pandemic restrictions (values <2% are plotted without labels).

Thirty percent (44/148) of respondents indicated that they were using telerehabilitation in clinical practice before the pandemic. This rose to 94% (138/147) during the period of COVID-19 restrictions and reduced to 82% (118/144) after restrictions were lifted. Only 3% (4/152) of the sample indicated that they had never provided telerehabilitation consultations (individual or group videoconferencing, or telephone consultations). Total telerehabilitation caseload rose to account for almost 47% of the total caseload during the period of restrictions but dropped substantially to 14% once restrictions were lifted, but still remained above the prepandemic level of 4% (Figure 1). This pattern was fairly consistent across areas of practice over the COVID-19 pandemic (Figure S1 in Multimedia Appendix 2).

**Figure 2.**
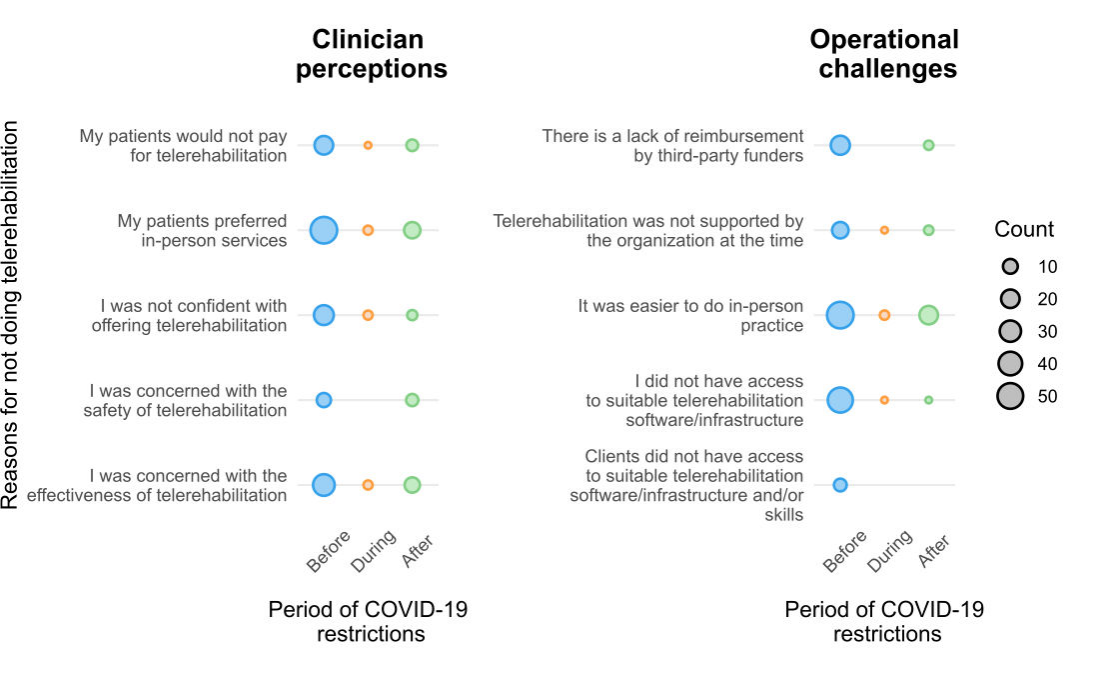
Reasons for not providing telerehabilitation throughout the phases of the COVID-19 pandemic, grouped by clinician perceptions and operational challenges. The area of circles represents the total count of respondents listing that reason for that point in time.

Reasons for not providing telerehabilitation consultations during each phase of the pandemic are provided in Figure 2. Across all 3 phases, the primary reasons were the perception that patients prefer in-person consultations (83/139, 60%) and that it was easier to do in-person consultations (55/139, 55%; Figure 2).

Before the pandemic, additional reasons for not offering telerehabilitation were primarily the perception that there was no need for telerehabilitation (57/104, 55%) or that physiotherapists did not have access to suitable telerehabilitation software or infrastructure (47/104, 45%; Table S1 in Multimedia Appendix 3). After restrictions were eased, the primary reasons for not providing telerehabilitation services were that respondents were concerned about the effectiveness of telerehabilitation (13/26, 50%) and did not like providing care via telerehabilitation (11/26, 42%; Table S1 in Multimedia Appendix 3).

### Shifts in Confidence, Effectiveness, and Satisfaction With Telerehabilitation

**Figure 3.**
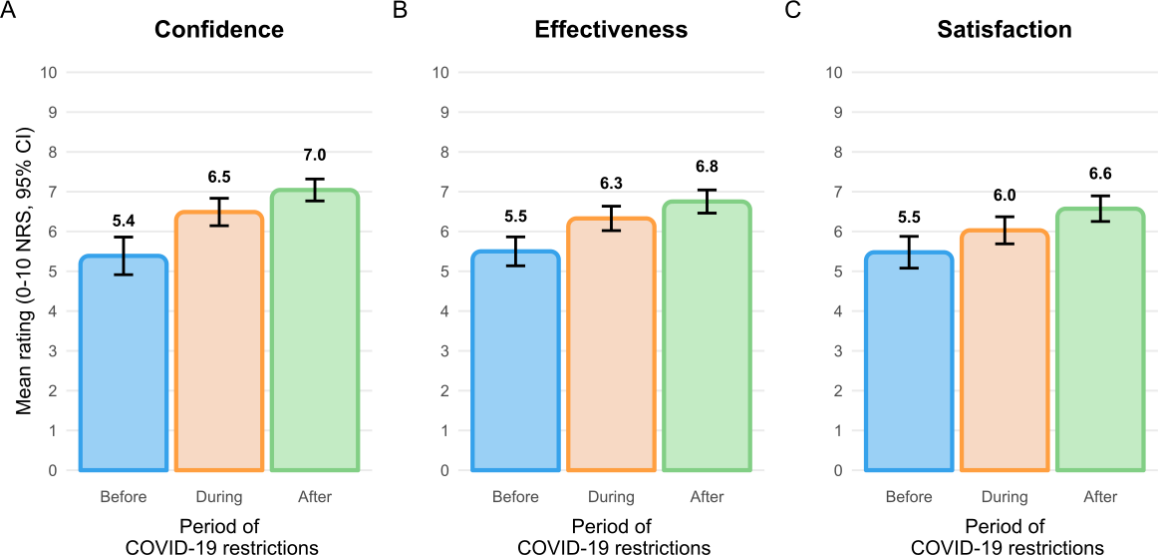
Participant ratings of (A) confidence, (B) perceived effectiveness, and (C) satisfaction with telerehabilitation across the pandemic. NRS: numerical rating scale.

Physiotherapist ratings of confidence in providing care via telerehabilitation, perceived effectiveness of telerehabilitation, and satisfaction with telerehabilitation progressively increased from before, during, to after restrictions associated with the COVID-19 pandemic (Figure 3). Almost 85% (120/142) of respondents indicated that providing telerehabilitation had become easier over time.

### Intended Versus Actual Telerehabilitation Use

**Figure 4.**
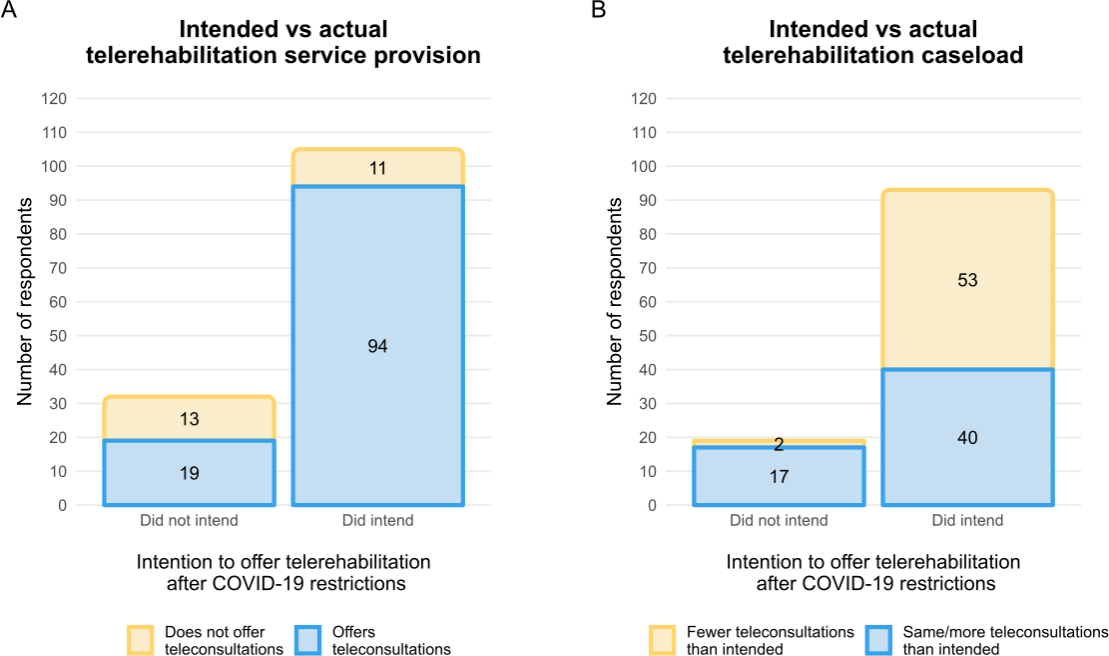
Intended versus actual telerehabilitation service provision and caseload. (A) Number of respondents who intended to offer telerehabilitation after easing of pandemic restrictions compared to whether they do offer telerehabilitation now. (B) Number of respondents who intended to offer telerehabilitation after easing of pandemic restrictions compared to whether the frequency of telerehabilitation met intentions.

Most respondents (105/137, 77%) intended to offer telerehabilitation after the easing of COVID-19 restrictions (Figure 4). Of these, only 10% (11/105) did not offer telerehabilitation despite intending to do so (Figure 4A). Primary reasons for not intending to offer telerehabilitation were because it was “easier to do in-person” (23/32, 72%) and because “patients prefer in-person” (22/32, 69%; Table S2 in Multimedia Appendix 3). Of those who did not intend to, more than half (19/32, 59%) did continue to offer telerehabilitation after restrictions were eased (Figure 4A). Approximately 50% of respondents who intended to continue offering telerehabilitation consultations (53/93) or who actually continued offering them (58/118) after restrictions were eased were providing fewer consultations than initially intended (Figure 4B). Respondents indicated that this was because patients “prefer in-person services” (44/58, 76%), “patient demand reduced more than expected” (32/38, 55%) and because it was “easier to do in-person consultations” (27/38, 47%; Table S3 in Multimedia Appendix 3). Primary reasons for continuing to offer telerehabilitation services included that telerehabilitation allowed physiotherapists to offer services to patients who would not usually be able to attend their clinic (84/118, 71%), that patients like the option of receiving care via telerehabilitation (76/118, 64%), and that patients find telerehabilitation convenient (74/118, 63%; Table S4 in Multimedia Appendix 3).

### Factors That Influence Telerehabilitation Use Postpandemic Restrictions

**Figure 5.**
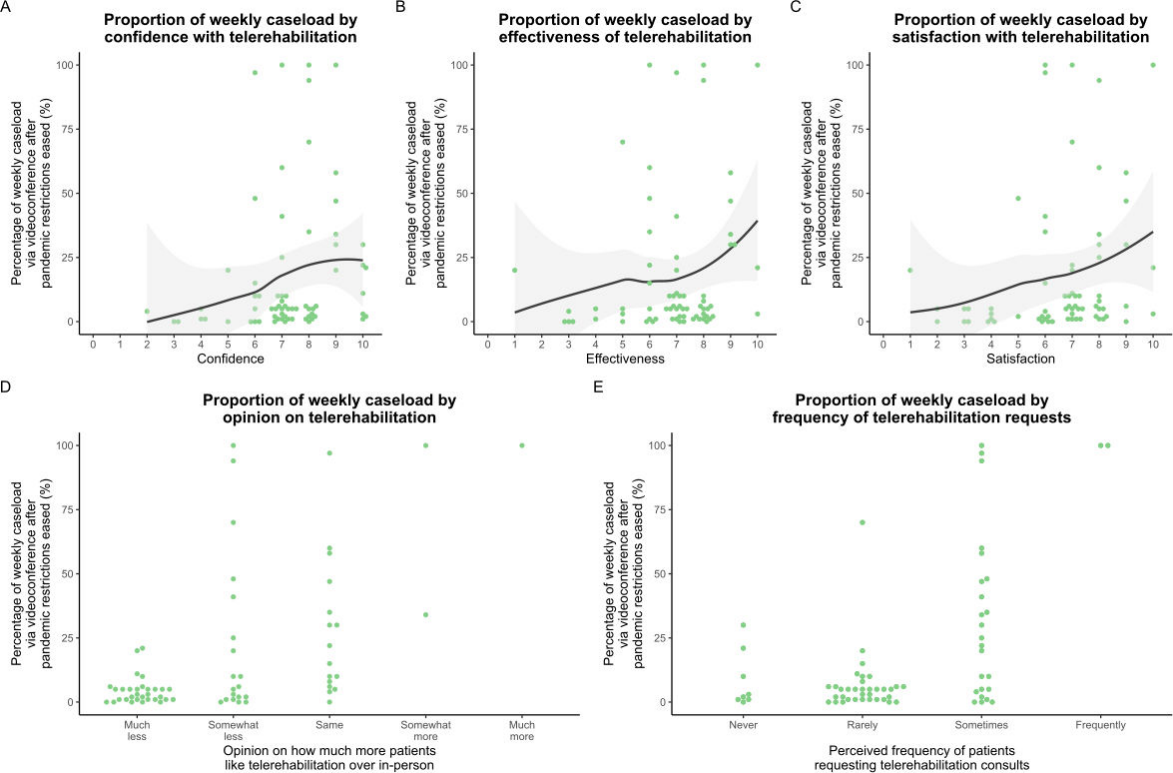
noTotal weekly telerehabilitation caseload versus (A) confidence, (B) effectiveness, (C) satisfaction, (D) patient liking for telerehabilitation, and (E) patient requests for telerehabilitation. Dark gray lines represent locally estimated scatterplot smoothing fits (using the whole span of the data) with 95% CIs (light gray shading).

Positive correlations were noted between a higher proportion of weekly caseload conducted via telerehabilitation and higher ratings of confidence in using telerehabilitation (Figure 5A), perceived effectiveness of telerehabilitation (Figure 5B), and satisfaction with telerehabilitation (Figure 5C).

Almost half of the respondents (69/142, 49%) indicated that patients were “rarely” requesting telerehabilitation since the easing of restrictions, and in the opinion of approximately half of the respondents (70/142, 49%), patients like telerehabilitation “much less than in-person consultations.” Physiotherapists who believed that patients liked telerehabilitation much less than in-person consultations appeared to have a lower proportion of their weekly caseload conducted via telerehabilitation (Figure 5D). Similarly, physiotherapists who reported that their patients requested telerehabilitation at least sometimes seemed more likely to have a higher proportion of weekly cases conducted via telehealth (Figure 5E).

The median (IQR) percentage of patients considered unsuitable for telerehabilitation by the respondents was 50.5% (50). Patient complexity and conditions requiring hands-on treatment were the primary reasons that patients were “often” considered unsuitable (Figure S2 in Multimedia Appendix 4 and Table S5 in Multimedia Appendix 3). Additional reasons respondents provided for deeming patients unsuitable are provided in Table S6 in Multimedia Appendix 3, with the largest proportion being patient preference for in-person consultations (6/30, 20%), physical examination being indicated (4/30, 13%), and complex patient presentations (6/30, 14%).

### Telerehabilitation Clinical Practice Considerations

Although only 5% (8/142) of respondents reported never experiencing technical issues themselves, most (120/142, 84%) indicated encountering these issues rarely or sometimes, and just 9% (14/142) experienced them often. Likewise, only one respondent (1/142, 1%) reported that their patients had never encountered technical issues, whereas the majority (112/142, 79%) reported that patients experienced technical issues rarely or sometimes, and 19% (29/142) reported that patients often experienced technical issues. When technical issues were encountered, 74% (105/142) reported only moderate or less disruption to the consultation. Only 5% (7/142) reported that technical issues were extremely disruptive, and just 4% (6/142) reported often having to cancel or reschedule appointments due to technical issues (Figure S3 in Multimedia Appendix 5). To support the delivery of telerehabilitation consultations, physiotherapists used text message reminders (109/142, 77%); written or digital educational material about the condition (67/142, 47%); and written instructions, diagrams, or booklets (63/142, 44%; Table S7 in Multimedia Appendix 3).

Almost three-quarters (104/142, 73%) of respondents indicated that they used similar parameters of care for telerehabilitation as for in-person consultations (eg, similar consultation frequency, duration, and similar content). For the respondents indicating that parameters of care were different (38/142, 27%), the primary reasons were that physical assessment or treatment was limited via telerehabilitation (15/38, 39%), consultations were shorter (10/38, 26%), and consultations were more focused on exercise or education (8/38, 21%). Additional reasons are provided in Table S8 in Multimedia Appendix 3. Similarly, most respondents (128/142, 90%) indicated that telerehabilitation consultations were about the same duration or shorter than in-person consultations (Table S9 in Multimedia Appendix 3) and that consultation frequency was “about the same” as in person (72/142, 51%) or less often than in person (53/142, 37%; Table S9 in Multimedia Appendix 3).

Considering the cost of telerehabilitation consultations, almost three-quarters of physiotherapists indicated that they charged “about the same” as an in-person consultation (104/142, 73%), with very few respondents (5/142, 4%) charging more than in-person consultations. Responses about the cost to the business of providing telerehabilitation were similar, with 50% (71/142) of respondents considering telerehabilitation to cost about the same and 36% (51/142) indicating that telerehabilitation consultations cost the business less than in-person consultations (Table S9 in Multimedia Appendix 3).

The median proportion of patients offered hybrid care in a current weekly caseload was 5% (minimum=0, Q1=1, Q3=20, maximum=100). In hybrid models of care, 42% (48/113) of physiotherapists indicated that patients typically receive many more in-person than telerehabilitation consultations, 27% (30/113) receive the same, and 18% (21/113) receive fewer in-person visits compared to telerehabilitation consultations (Table S9 in Multimedia Appendix 3). Other ways in which telerehabilitation models of care differ from in-person models were coded qualitatively and provided in Table S10 in Multimedia Appendix 3. When respondents used a hybrid model, 27% (8/27) offered telerehabilitation only after an initial in-person consult, 15% (4/27) described limiting the physical assessment or treatment component of consultations, and 11% (3/27) said telerehabilitation consultations in hybrid models had a greater case management focus (3/27, 11%).

Inductive content analysis of free-text responses identified four key themes that reflected respondents’ perspectives on using telerehabilitation in clinical practice postpandemic (Table S11 in Multimedia Appendix 3): (1) concerns about telerehabilitation, (2) perceived benefits of telerehabilitation, (3) how telerehabilitation is used in practice, and (4) physiotherapists’ willingness to provide telerehabilitation services.

#### Theme 1: Concerns About Telerehabilitation (n=28)

Physiotherapists expressed a range of concerns about the suitability and practicality of telerehabilitation in postpandemic physiotherapy care. The most commonly reported issue was that clients prefer or actively seek in-person consultations (n=12, 43%). For example, one participant said that despite telerehabilitation remaining available for their patients, they “*often prefer face-to-face*” and that “*people wanted to revert back to the ‘usual’ ways and leave the changes of COVID behind them moving forward once restrictions eased*” (musculoskeletal physiotherapist). Some respondents (n=4, 14%) emphasized that telerehabilitation is not suitable for all clients, particularly those with complex conditions, communication difficulties, or low digital literacy, and that they were selecting suitable clients for telerehabilitation, and “*not offering [it] for those not ‘tech-savvy*’” (neurological physiotherapist). Concerns were also raised about the limitations of assessment via videoconference (n=2, 7%) and challenges related to internet connectivity and software reliability (n=3, 11%). Additional issues included payment and reimbursement barriers (n=2, 7%), difficulties building rapport remotely (n=2, 7%), and reduced referrals and attendance for telerehabilitation compared to in-person care.

#### Theme 2: Perceived Benefits of Telerehabilitation (n=20)

Despite concerns, respondents acknowledged several advantages of using telerehabilitation in their clinical practice postpandemic restrictions. The most frequently cited benefit was that telerehabilitation improved patient access to care, particularly for those in rural or remote areas or those with difficulties with travel or limited time (n=8, 40%). One musculoskeletal physiotherapist said that telerehabilitation “*has made physiotherapy much more accessible to a wider population and allows people greater flexibility with appointments*.” Participants also noted an increased acceptance of telerehabilitation (among patients and providers; n=6, 30%), with some suggesting that it “*has become common practice now*” (musculoskeletal physiotherapist) and that it can be effective for certain presentations (eg, chronic musculoskeletal conditions; n=3, 15%), for supporting patient self-management (n=2, 10%) and providing greater flexibility in service delivery (n=1, 5%).

#### Theme 3: How Telerehabilitation Is Used in Practice (n=8)

Participants described integrating telerehabilitation into their clinical practice for subsequent consultations following initial in-person visits (n=2, 25%), for triaging (n=1, 12.5%) and case management (n=1, 12.5%), and as a tool for exercise prescription (n=1, 12.5%). Some participants (n=2, 25%) indicated that videoconferencing was preferred over telephone, and 1 (12.5%) participant noted that at times additional support is required at the patient end to effectively deliver telerehabilitation services. One cardiorespiratory, hospital-based physiotherapist described that telerehabilitation “*consultations have been effective in triaging patients and determining the appropriate level of care required*,” whereas another described that telerehabilitation “*has been a great option for follow-up appointments, especially when you have already build rapport with patients...[and it]...has been a great way to check in with people who have busy schedules or live far away and find it difficult coming in*” (pelvic health and musculoskeletal physiotherapist).

#### Theme 4: Physiotherapists' Willingness to Provide Telerehabilitation Services (n=23)

Many participants (n=16, 70%) were willing to continue providing telerehabilitation services, driven by the perceived benefits and uses of telerehabilitation. For example, one private practice, musculoskeletal and mental health physiotherapist said that “*for the provision of exercise and movement based interventions, telerehabilitation has worked better than in-person as it provides easier access to more people given I live in a regional area*.” Despite this willingness, some participants expressed low satisfaction with telerehabilitation (n=2, 9%) or a preference for in-person consultations (n=2, 9%). For example, a sports, exercise, and musculoskeletal physiotherapist working in private practice said that despite telerehabilitation “*opening up my practice to lots of different people around Australia and internationally... I still prefer to consult in-person*...” The need for ongoing education about the utility of telerehabilitation in physiotherapy was noted (n=1, 4%), despite the perception that education about how to deliver telerehabilitation had improved during the pandemic (n=1, 4%).

## Discussion

### Principal Findings

Despite an initial increase due to the COVID-19 pandemic restrictions and physiotherapists’ intentions to continue offering telerehabilitation services, many physiotherapists were offering fewer telerehabilitation consultations than anticipated once restrictions were lifted. This was primarily due to a preference for in-person consultations, concerns about the effectiveness of telerehabilitation, and the perception that physiotherapy consultations are easier to conduct in person.

International postpandemic data across both physiotherapy and other health services show a similar “peak-to-plateau” pattern, where telehealth usage increased substantially during restrictions before falling and stabilizing at a lower level rather than returning to prepandemic levels. In a Polish national dataset, telehealth in both outpatient health and rehabilitation services (excluding mental health) rose from prepandemic levels near zero to peak in 2020 before subsequently stabilizing at approximately one-fifth and one-third of their respective peak volumes [25]. Similarly, musculoskeletal physical therapists in the United States reported reduced telerehabilitation usage postpandemic, albeit at levels higher than prepandemic [22]. Likewise, across the US health system, overall telehealth usage peaked in 2020 and then declined but stabilized by 2023 [26,27]. Notably, however, telehealth usage was better sustained for services less dependent on hands-on care (eg, behavioral health and psychiatry) and in states where policies were put in place to ensure payment parity with comparable in-person services [26].

Although the perceived effectiveness of telerehabilitation had increased over the course of the pandemic, more than half of participants still identified concerns about the effectiveness of telerehabilitation as a primary reason to stop offering telerehabilitation consultations once able to resume in-person services. This is consistent with other studies conducted during the pandemic, where physiotherapists indicated concerns about the effectiveness of telerehabilitation for physiotherapy assessment and/or management [13,28].

Research indicates that outcomes for telerehabilitation are the same, if not better, than in-person physiotherapy for a range of conditions. For example, systematic reviews and randomized controlled trials in musculoskeletal, cardiac, and pulmonary populations demonstrate the noninferiority of telerehabilitation [29] and good validity for assessment conducted via telerehabilitation [30]. Physiotherapists’ perceptions may be centered around occupational self-efficacy [31] or their own personal clinical experience of “effectiveness” rather than evidence of effectiveness in the published literature. However, our results suggest that physiotherapists were not concerned about their own ability to deliver services via telerehabilitation. Both perceived satisfaction with telerehabilitation and confidence in delivering telerehabilitation trended upward from the prepandemic to the postpandemic period, and “I was not confident with telerehabilitation” was not a key factor in our findings after restrictions were lifted (3/26, 12%) . However, large proportions of respondents who did not offer telerehabilitation at this stage said it was easier to do in-person consultations instead (20/26, 70%); they were concerned with the effectiveness of telerehabilitation (13/26, 50%), and they did not like providing care via telerehabilitation (11/26, 42%). These findings suggest that there are additional factors influencing physiotherapists’ perceptions about the superiority of “hands-on” or “in-person” physiotherapy [32-34] that have not been comprehensively explored, such as the professional identity of a physiotherapist [31,35].

A qualitative study describing a successful, rapid transition to telerehabilitation during the pandemic challenges the perception that physiotherapy requires “hands-on” approaches and needs to be in person [36]. This study identified that physiotherapists’ readiness and willingness to modify their approach influenced the success of telerehabilitation. In our study, physiotherapists preferred in-person consultations themselves and perceived that their patients also preferred in-person consultations, which is likely to influence whether they offer telerehabilitation to patients. While systematic reviews suggest that patient satisfaction with telerehabilitation is comparable to and often higher than in-person care [37,38], many patients report a preference for in-person physiotherapy if given a choice [37,39]. Although physiotherapists might have thought during the pandemic that patient demand for telerehabilitation would remain (eg, explaining their intention to offer it), if patient demand for it decreased (as 55% of our sample indicated), physiotherapists would likely perceive that patients prefer in-person care (and indeed 76% of our sample did).

Clinician preferences for providing in-person physiotherapy have also been explored and reported on in the literature. Despite high levels of clinician satisfaction when providing telerehabilitation in clinical trials [40,41], this does not appear to be the case for *in-practice* preference for, or satisfaction with, telerehabilitation [21,22,28]. Although satisfaction and confidence with telerehabilitation increased over time, participants in this study still perceived in-person physiotherapy to be easier. The rigorous planning or structured training required for telerehabilitation delivery in a randomized clinical trial, rather than day-to-day clinical practice, may explain this difference in perceptions, highlighting a need for training specific to the clinical implementation of telerehabilitation. Studies examining barriers to implementing telerehabilitation in routine physiotherapy practice consistently identify insufficient training for conducting telerehabilitation consultations as a primary concern [42]. To address these challenges, international clinical practice guidelines provide evidence-based recommendations and strategies for overcoming barriers, guiding the training of clinicians and facilitating effective implementation of telerehabilitation into physiotherapy practice [43].

Clinicians have long identified the technological illiteracy of clients as a barrier to the adoption of telerehabilitation in physiotherapy [42]. Despite advances in technology infrastructure, when transitioning to telerehabilitation during the COVID-19 pandemic period, clinicians still identified “technology concerns” (including clinician concerns about client ability to use technology) as a barrier to telerehabilitation use in clinical practice [28,34,36,42,44]. In our study, concerns about technical issues or patients being unable to use or access technology were not identified as primary reasons physiotherapists determined patients were unsuitable for telerehabilitation. Additionally, technical issues were only slightly or moderately disruptive to consultations. This is consistent with findings from an evaluation of consultations delivered in a randomized controlled trial, which found that technical issues occurred but were infrequent and minimally disruptive [45]. This could potentially be because, at the time data were collected for this study (2023), physiotherapists and clients had greater experience with and exposure to the technology required for telerehabilitation and had become more comfortable over time [46]. In other studies where data were collected earlier in the pandemic, it is possible that fewer people were familiar with telerehabilitation technology, hence it being a bigger barrier to delivering telerehabilitation services at the time [20,21,36].

### Strengths and Limitations

The findings of this study should be interpreted with the following limitations in mind. First, this was a small convenience sample, and findings may have been skewed by self-selection bias (with those with strong opinions, either positive or negative, electing to complete the survey). Second, we asked participants to recall what they were doing before and during the pandemic several years after the fact. Therefore, it should be acknowledged that participants’ responses may have been influenced by recall bias. However, our data pertaining to before and during the pandemic were consistent with other studies conducted during the pandemic and their intentions to continue (eg, 69% in our study said that during the pandemic, they intended to offer telerehabilitation after restrictions were eased). In a study by Bennell et al [20], 81% intended to continue offering telerehabilitation consultations after the pandemic, and in a study by Peng et al [28], 55% and 68% intended to continue offering phone and videoconferencing, respectively. If the opportunity arises (ie, another period of restrictions to in-person consultations), researchers should consider using prospective study designs. Moreover, because our survey encompassed both phone calls and videoconferencing, our findings may not reflect modality-specific differences in perceptions reported elsewhere [28]. This survey also only sampled physiotherapists operating within Australia’s health care system, so its findings may not fully translate to other countries with different telerehabilitation policies, funding models, or cultural attitudes toward remote care. Finally, due to an error, questions about confidence, satisfaction, and perceived effectiveness after the pandemic restrictions were eased were misworded and instead asked about experiences during the pandemic. It is likely that, given that all questions before this were about easing restrictions, most respondents still answered according to the intention of the question, but we cannot discount that some answered more literally, thereby skewing the data.

### Conclusions

Although telerehabilitation use surged with pandemic restrictions, it has subsequently decreased significantly, with telerehabilitation accounting for only a small proportion of the total caseload. Despite increased confidence and satisfaction with telerehabilitation, clinician preference, and physiotherapists’ perceptions of patient preference for in-person care, reduced demand and the ease of in-person practice influence the use of telerehabilitation postrestrictions and suggest persistent barriers to frequent use. Addressing these barriers is crucial to enhance the long-term viability and effectiveness of telerehabilitation physiotherapy in Australia.

## Supplementary material

10.2196/81008 Multimedia Appendix 1 Survey.

10.2196/81008 Multimedia Appendix 2 Telerehabilitation caseload over the COVID-19 pandemic for each physiotherapy area of practice.

10.2196/81008 Multimedia Appendix 3 Detailed quantitative (n, %) and qualitative survey findings on physiotherapists’ telerehabilitation practice across COVID-19 restrictions: reasons for not offering, reducing, ceasing, or continuing telerehabilitation; reasons patients were considered unsuitable and the resources used to support videoconferencing; differences in consultation parameters and hybrid models of care compared with in-person practice; and postrestriction perspectives and willingness to provide telerehabilitation.

10.2196/81008 Multimedia Appendix 4 Patient suitability for telerehabilitation.

10.2196/81008 Multimedia Appendix 5 Technical issues.

## Abbreviations

CROSS: Checklist for Reporting of Survey Studies
