# Supplementary material for: Telerehabilitation Trends in Australian Physiotherapy and an Exploration of Factors That Influence Use After COVID-19 Restrictions: Qualitative Content Analysis
Source: JMIR Rehabil Assist Technol. 2026 Jan 27;13:e81008. doi: 10.2196/81008 (PMC12844842; doi:10.2196/81008)
Supplement: Multimedia Appendix 1 [file rehab-v13-e81008-s001.docx]

Appendix 1: Survey

Start of Block: Information + Consent

Q1
**Participant Information Sheet**

**Research Title:** A post-COVID exploration of telerehabilitation in physiotherapy practice

**Researcher(s):**
Dr Megan Ross, RECOVER Injury Research Centre ([m.ross@uq.edu.au](mailto:m.ross@uq.edu.au))

Dr Joshua Simmich, RECOVER Injury Research Centre ([j.simmich@uq.edu.au](mailto:j.simmich@uq.edu.au))

Dr Belinda Lawford, The University of Melbourne ([belinda.lawford@unimelb.edu.au](mailto:belinda.lawford@unimelb.edu.au))

Prof Rana Hinman, The University of Melbourne ([ranash@unimelb.edu.au](mailto:ranash@unimelb.edu.au))

Prof Kimberley Bennell, The University of Melbourne ([k.bennell@unimelb.edu.au](mailto:k.bennell@unimelb.edu.au))

Prof Trevor Russell, RECOVER Injury Research Centre ([t.russell@uq.edu.au](mailto:t.russell@uq.edu.au))

**Please read the following and provide consent to participate at the bottom of this form**

Thank you for your interest in participating in this research project. Please read the following information about the project, so that you can decide whether you would like to take part in this research. Please feel free to ask any questions you might have about our involvement in the project.

If you decide to participate in this research, please keep in mind that your participation is voluntary. If you do not wish to take part, you do not have to. If you decide to take part and later change your mind, you are free to stop at any time, and you would not need to give any explanation for your decision to stop participating. If you choose to stop participating, your data will not be used in the research. Your decision whether you take part, or not to take part, or to take part and then withdraw, will not affect your relationship with the University of Queensland.

**What is this research about?**

This research project aims to explore physiotherapists’ use of telerehabilitation in clinical practice after the COVID-19 pandemic.

**What will I need to do?**

If you agree to participate in this study you will be asked to complete an online survey. The survey will be completed in two (2) parts. In Part 1, you will be asked to provide some information about yourself, your clinical practice and your experience with telerehabilitation. It is anticipated that this part will take a maximum of 5 minutes to complete. In Part 2, you will be asked a range of questions about your use of telerehabilitation after restrictions were eased. It is anticipated that this will take approximately 20 minutes to complete.

**What are the possible benefits of taking part?**

While there is no direct benefit to you for participating in this study, your participation can help to advance the knowledge in the field of physiotherapist-delivered telerehabilitation consultations. The results of this study can help better understand physiotherapists use of and preferences for delivering telerehabilitation services. If you opt in, you can **go in the draw to win a $1000 gift voucher**.

**What are the possible risks and disadvantages of taking part?**

There are no known risks associated with participating in this study. However, some participants may experience discomfort or inconvenience from completing the survey.

**What will happen to the information about me?**

Your responses will be kept confidential and anonymous. No identifiable information is being collected, unless you indicate you would like to receive a copy of the published research paper, wish to go in the prize draw, or indicate that you are happy to be contacted for future related research. This information will be stored separately from your responses to the survey questions. All data will be stored securely on The University of Queensland Research Data Management System and only the research team will have access to the data collected.

**What will happen if I decide to withdraw?**

Your participation in this research is voluntary and you are free to withdraw from the research anytime without needing to provide any explanation, and you would not receive any penalty or bias as a result of your withdrawal. Should you decide to withdraw, you can indicate if all the information collected from/about you should be destroyed and not used in the research.

**Can I hear about the results of this research?**

Results of this research will be made available to you upon request. However, please note that the results will be presented in an aggregated form, and individual responses will not be identified.

It is anticipated that the results of this research project will be published and/or presented in a variety of forms. In any publication and/or presentation, information will be provided in such a way that you cannot be identified.

**Who can I contact if I have any concerns about the project?**

This study adheres to the Guidelines of the ethical review process of The University of Queensland and the National Statement on Ethical Conduct in Human Research. Whilst you are free to discuss your participation in this study with the researcher contactable on m.ross@uq.edu.au if you would like to speak to an officer of the University not involved in the study, you may contact the Ethics Coordinator on +617 3365 3924 / +617 3443 1656 or email [humanethics@research.uq.edu.au](mailto:humanethics@research.uq.edu.au).

This research Ethics ID number: 2023/HE001802

Q2 This statement confirms that I have read and understood the information provided, and I voluntarily agree to participate in this research project.

- I agree to participate in this research project. (1)

End of Block: Information + Consent

Start of Block: Screening

Q3 Are you an APHRA registered physiotherapist currently practicing in an Australian private practice or community setting?

- Yes (1)
- No (2)

Skip To: End of Survey If Are you an APHRA registered physiotherapist currently practicing in an Australian private practic... = No

End of Block: Screening

Start of Block: Demographics

Q4 Thank you for taking the time to participate in this research project. It should take no more than 20 minutes for you to complete. First, you will be asked to complete some details about yourself, followed by some questions about your use of telehealth in clinical practice. For the purposes of this questionnaire, we will assume the COVID-19 pandemic was during the years 2020-2021 when most restrictions were in place. Throughout this survey, the term telehealth will be used to refer to individual or group-based consultations that are delivered either via videoconferencing or the telephone.

| Page Break |  |
| --- | --- |

Q5 With which gender do you most identify?

- Man (1)
- Woman (2)
- Non-binary / third gender (3)
- Prefer not to say (4)
- Prefer to self describe: (5) __________________________________________________

Q6 State / territory of your practice:

- New South Wales (NSW) (1)
- Queensland (QLD) (2)
- South Australia (SA) (3)
- Tasmania (TAS) (4)
- Victoria (VIC) (5)
- Western Australia (WA) (6)
- Australian Capital Territory (ACT) (7)
- Jervis Bay Territory (JBT) (8)
- Northern Territory (NT) (9)
- Ashmore and Cartier Islands (10)
- Christmas Island (11)
- Cocos (Keeling) Islands (12)
- Coral Sea Islands (13)
- Heard Island and McDonald Islands (14)
- Norfolk Island (15)

| 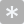 |
| --- |

Q7 Postcode of practice:

________________________________________________________________

Q8 Do you have any post graduate qualifications?

- Yes (1)
- No (2)

Q9 Please specify your highest level of completed education.

- PhD (1)
- Masters by research (2)
- Masters by course work (3)
- Postgraduate diploma (4)
- Bachelor's degree (5)
- Associate degree (6)
- Other, please specify: (7) __________________________________________________

Q78 If you are human please enter 99 in the box below

________________________________________________________________

Q10 Have you had any prior training specifically in the delivery of telehealth?

- No (1)
- Yes, < 6 months ago (2)
- Yes, between 6 and 12 months ago (3)
- Yes, between 12 months and 2 years ago (4)
- Yes, between 2 and 3 years ago (5)
- Yes, longer than 3 years ago (6)

Display this question:

If Have you had any prior training specifically in the delivery of telehealth? != No

| 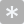 |
| --- |

Q11 Please specify the number of hours of training specifically in the delivery of telehealth:

________________________________________________________________

Display this question:

If Have you had any prior training specifically in the delivery of telehealth? != No

Q12 Did the training include a practical component (e.g. used technology under guidance of an educator to provide a real or simulated consultation)?

- No (1)
- Yes, please provide details: (2) __________________________________________________

Q13 Please describe your physiotherapy practice(s) (you may tick more than one):

- Private practice (primary care) (1)
- Private hospital (2)
- Community health centre (3)
- Public health outpatient centre (4)
- Other, please specify: (5) __________________________________________________

| 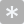 |
| --- |

Q14 Predominant focus of your usual clinical caseload (tick up to 2):

- Musculoskeletal/orthopaedic (1)
- Sports and exercise (2)
- Paediatric (3)
- Neurology (4)
- Cardiorespiratory (5)
- Gerontology (6)
- Occupational health (7)
- Aquatic (8)
- Women’s, men’s and pelvic health (9)
- Cancer, palliative care and lymphoedema (10)
- Mental health (11)

Q15 Have you (personally) **ever** offered any of these telehealth services (tick all that apply):

- Individual one-to-one videoconferencing consultations (1)
- Group classes via videoconferencing (2)
- Telephone consultations (3)
- None of the above (4)

Display this question:

If Have you (personally) ever offered any of these telehealth services (tick all that apply): = None of the above

Q16 Can you explain the reasons that you have not engaged in telehealth practice (tick all that apply):

- My patients would not pay for telehealth (1)
- There is a lack of reimbursement by third party funders (2)
- I did not have access to suitable telehealth software/infrastructure (3)
- It is easier to do in-person practice (4)
- I am not confident with offering telehealth (5)
- I am concerned with the safety of telehealth (6)
- I am concerned with the effectiveness of telehealth (7)
- The business costs of offering telehealth was too high (8)
- My patients prefer in-person services (9)
- Other, please describe: (10) __________________________________________________

Display this question:

If Have you (personally) ever offered any of these telehealth services (tick all that apply): = None of the above

Q17 Please describe any circumstances that might make you consider offering telehealth services in the future.

________________________________________________________________

Display this question:

If Have you (personally) ever offered any of these telehealth services (tick all that apply): = None of the above

Q18 Thank you for taking the time to complete this survey.  Would you like to be entered into a draw to receive $1000 gift voucher in appreciation of completing this survey?

- No (1)
- Yes, please provide your Name and Email address: (2) __________________________________________________

Skip To: End of Survey If Thank you for taking the time to complete this survey.  Would you like to be entered into a draw... , No Is Displayed

End of Block: Demographics

Start of Block: Prior to COVID

Q19 Prior to the COVID-19 pandemic, did you provide consultations via telehealth?

- No (1)
- Yes (2)

Display this question:

If Prior to the COVID-19 pandemic, did you provide consultations via telehealth? = No

Q20 Why did you not engage in telehealth practice prior to COVID-19 (tick all that apply):

- I didn’t see the need (1)
- My patients would not pay for telehealth (2)
- There is a lack of reimbursement by third party funders (3)
- I did not have access to suitable telehealth software/infrastructure (4)
- It was easier to do in-person practice (5)
- I was not confident with offering telehealth (6)
- I was concerned with the safety of telehealth (7)
- I was concerned with the effectiveness of telehealth (8)
- The business costs of offering telehealth was too high (9)
- My patients preferred in-person services (10)
- Other (11) __________________________________________________

Display this question:

If Prior to the COVID-19 pandemic, did you provide consultations via telehealth? = No

Q21 Are there any other reasons why you did not offer telehealth consultations prior to the pandemic?

- No (1)
- Yes, please describe: (2) __________________________________________________

Skip To: End of Block If Are there any other reasons why you did not offer telehealth consultations prior to the pandemic? , No Is Displayed

Q22 In any given week prior to the COVID-19 pandemic, what proportion of your weekly patient caseload was provided as **individual videoconferencing consultations**:  *For example, if 70% of your caseload was provided as in-person, 15% was provided as individual consultations via videoconferencing, 10% was provided as group-based videoconferencing and 5% was provided as telephone, you should answer:* *15% for "individual videoconferencing consultations"*

|  | Not Applicable |
| --- | --- |

|  | 0 | 10 | 20 | 30 | 40 | 50 | 60 | 70 | 80 | 90 | 100 |
| --- | --- | --- | --- | --- | --- | --- | --- | --- | --- | --- | --- |

| Individual videoconferencing consultations () | 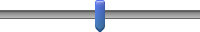 |
| --- | --- |

Q23 In any given week prior to the COVID-19 pandemic, what proportion of your weekly patient caseload was provided as **group-based videoconferencing consultations**:  *For example, if 70% of your caseload was provided as in-person, 15% was provided as individual consultations via videoconferencing, 10% was provided as group-based videoconferencing and 5% was provided as telephone, you should answer:* *10% for "group-based videoconferencing consultations" and*

|  | Not Applicable |
| --- | --- |

|  | 0 | 10 | 20 | 30 | 40 | 50 | 60 | 70 | 80 | 90 | 100 |
| --- | --- | --- | --- | --- | --- | --- | --- | --- | --- | --- | --- |

| Group-based videoconferencing consultations () | 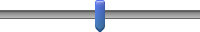 |
| --- | --- |

Q24 In any given week prior to the COVID-19 pandemic, what proportion of your weekly patient caseload was provided as **telephone consultations**:  *For example, if 70% of your caseload was provided as in-person, 15% was provided as individual consultations via videoconferencing, 10% was provided as group-based videoconferencing and 5% was provided as telephone, you should answer:* *5% for "telephone consultations".*

|  | Not Applicable |
| --- | --- |

|  | 0 | 10 | 20 | 30 | 40 | 50 | 60 | 70 | 80 | 90 | 100 |
| --- | --- | --- | --- | --- | --- | --- | --- | --- | --- | --- | --- |

| Telephone consultations () | 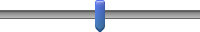 |
| --- | --- |

Q25 Prior to the COVID-19 pandemic, what was your level of confidence providing physiotherapy consultations using telehealth?

- Not at all confident: 1 (1)
- 2 (2)
- 3 (3)
- 4 (4)
- 5 (5)
- 6 (6)
- 7 (7)
- 8 (8)
- 9 (9)
- Extremely confident: 10 (10)

Q26 Prior to the COVID-19 pandemic, how effective did you find the physiotherapy care you provided to patients via telehealth?

- Not at all effective: 1 (1)
- 2 (2)
- 3 (3)
- 4 (4)
- 5 (5)
- 6 (6)
- 7 (7)
- 8 (8)
- 9 (9)
- Extremely effective: 10 (10)

Q27 Prior to the COVID-19 pandemic, how satisfied were you with the physiotherapy care you provided to patients via telehealth?

- Not at all satisfied: 1 (1)
- 2 (2)
- 3 (3)
- 4 (4)
- 5 (5)
- 6 (6)
- 7 (7)
- 8 (8)
- 9 (9)
- Extremely satisfied: 10 (10)

End of Block: Prior to COVID

Start of Block: During COVID restrictions (2020-2022)

Q28 During the COVID-19 pandemic restrictions, did you provide consultations via telehealth?

- No (1)
- Yes (2)

Display this question:

If During the COVID-19 pandemic restrictions, did you provide consultations via telehealth? = No

Q29 Why did you not engage in telehealth during the COVID-19 restrictions (tick all that apply):

- My practice closed during this period (1)
- My practice decided not to offer telehealth consultations (2)
- I didn’t see the need (3)
- My patients would not pay for telehealth (4)
- There is a lack of reimbursement by third party funders (5)
- I did not have access to suitable telehealth software/infrastructure (6)
- It was easier to do in-person practice (7)
- I was not confident with offering telehealth (8)
- I was concerned with the safety of telehealth (9)
- I was concerned with the effectiveness of telehealth (10)
- The business costs of offering telehealth was too high (11)
- My patients preferred in-person services (12)

Display this question:

If During the COVID-19 pandemic restrictions, did you provide consultations via telehealth? = No

Q30 Are there any other reasons why you did not offer telehealth consultations during the pandemic?

- No (1)
- Yes, please describe: (2) __________________________________________________

Skip To: End of Block If Are there any other reasons why you did not offer telehealth consultations during the pandemic? , No Is Displayed

Q31 In any given week during the COVID-19 pandemic, what proportion of your weekly patient caseload was provided as **individual videoconferencing consultations**:  *For example, if 70% of your caseload was provided as in-person, 15% was provided as individual consultations via videoconferencing, 10% was provided as group-based videoconferencing and 5% was provided as telephone, you should answer:* *15% for "individual videoconferencing consultations"*

|  | Not Applicable |
| --- | --- |

|  | 0 | 10 | 20 | 30 | 40 | 50 | 60 | 70 | 80 | 90 | 100 |
| --- | --- | --- | --- | --- | --- | --- | --- | --- | --- | --- | --- |

| Individual videoconferencing consultations () | 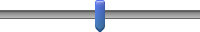 |
| --- | --- |

Q32 In any given week during the COVID-19 pandemic, what proportion of your weekly patient caseload was provided as **group-based videoconferencing consultations**:  *For example, if 70% of your caseload was provided as in-person, 15% was provided as individual consultations via videoconferencing, 10% was provided as group-based videoconferencing and 5% was provided as telephone, you should answer:* *10% for "group-based videoconferencing consultations"*

|  | Not Applicable |
| --- | --- |

|  | 0 | 10 | 20 | 30 | 40 | 50 | 60 | 70 | 80 | 90 | 100 |
| --- | --- | --- | --- | --- | --- | --- | --- | --- | --- | --- | --- |

| Group-based videoconferencing consultations () | 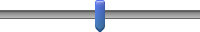 |
| --- | --- |

Q33 In any given week during the COVID-19 pandemic, what proportion of your weekly patient caseload was provided as **telephone consultations**:  *For example, if 70% of your caseload was provided as in-person, 15% was provided as individual consultations via videoconferencing, 10% was provided as group-based videoconferencing and 5% was provided as telephone, you should answer:* *5% for "telephone consultations".*

|  | Not Applicable |
| --- | --- |

|  | 0 | 10 | 20 | 30 | 40 | 50 | 60 | 70 | 80 | 90 | 100 |
| --- | --- | --- | --- | --- | --- | --- | --- | --- | --- | --- | --- |

| Telephone consultations () | 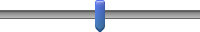 |
| --- | --- |

Q34 During the COVID-19 pandemic, what was your level of confidence providing physiotherapy consultations using telehealth?

- Not at all confident: 1 (1)
- 2 (2)
- 3 (3)
- 4 (4)
- 5 (5)
- 6 (6)
- 7 (7)
- 8 (8)
- 9 (9)
- Extremely confident: 10 (10)

Q35 During the COVID-19 pandemic, how effective did you find the physiotherapy care you provided to patients via telehealth?

- Not at all effective: 1 (1)
- 2 (2)
- 3 (3)
- 4 (4)
- 5 (5)
- 6 (6)
- 7 (7)
- 8 (8)
- 9 (9)
- Extremely effective: 10 (10)

Q36 During the COVID-19 pandemic, how satisfied were you with the physiotherapy care you provided to patients via telehealth?

- Not at all satisfied: 1 (1)
- 2 (2)
- 3 (3)
- 4 (4)
- 5 (5)
- 6 (6)
- 7 (7)
- 8 (8)
- 9 (9)
- Extremely satisfied: 10 (10)

| Page Break |  |
| --- | --- |

Q37 During the pandemic, did you plan to continue offering telehealth consultations into the future, after the pandemic was over?

- No (1)
- Yes (2)

Display this question:

If During the pandemic, did you plan to continue offering telehealth consultations into the future,... = No

Q38 Can you explain the reasons why you planned to stop offering telehealth after the easing of the pandemic restrictions (tick all that apply):

- I did not like providing care via telehealth (1)
- The administrative burden of offering telehealth in addition to in-person consults is too high (2)
- My practice decided to not offer telehealth consultations. (3)
- My patients would not pay for telehealth (4)
- There is a lack of reimbursement by third party funders (5)
- I did not have access to suitable telehealth software/infrastructure (6)
- It was easier to do in-person consultations (7)
- I was not confident with offering telehealth (8)
- I was concerned with the safety of telehealth (9)
- I was concerned with the effectiveness of telehealth (10)
- The business cost of offering telehealth was too high (11)
- My patients prefer in-person services (12)

Display this question:

If During the pandemic, did you plan to continue offering telehealth consultations into the future,... = No

Q39 Are there any other reasons why planned to not offer telehealth after the easing of the pandemic restrictions?

- No (1)
- Yes, please describe: (2) __________________________________________________

End of Block: During COVID restrictions (2020-2022)

Start of Block: After COVID Restrictions (from 2022)

Q40 After the COVID-19 pandemic restrictions were lifted (since 2022), have you provided consultations via telehealth?

- No (1)
- Yes (2)

Display this question:

If After the COVID-19 pandemic restrictions were lifted (since 2022), have you provided consultation... = No

Q41 Why did you not engage in telehealth since the easing of COVID-19 restrictions (tick all that apply):

- I did not like providing care via telehealth (1)
- The administrative burden of offering telehealth in addition to in-person consults is too high (2)
- My practice decided to not offer telehealth consultations (3)
- My patients would not pay for telehealth (4)
- There is a lack of reimbursement by third party funders (5)
- I did not have access to suitable telehealth software/infrastructure (6)
- It was easier to do in-person practice (7)
- I was not confident with offering telehealth (8)
- I was concerned with the safety of telehealth (9)
- I was concerned with the effectiveness of telehealth (10)
- The business costs of offering telehealth was too high (11)
- My patients prefer in-person services (12)

Display this question:

If After the COVID-19 pandemic restrictions were lifted (since 2022), have you provided consultation... = No

Q42 Are there any other reasons why you have not engaged in telehealth since the easing of COVID-19 restrictions?

- No (1)
- Yes, please describe: (2) __________________________________________________

Skip To: End of Block If Are there any other reasons why you have not engaged in telehealth since the easing of COVID-19 r... , No Is Displayed

Q43 Why have you continued to offer telehealth since the easing of COVID-19 restrictions (tick all that apply)?

- I like providing care via telehealth (1)
- I find that telehealth is effective (2)
- My patients like the option of receiving care via telehealth (3)
- My patients find telehealth convenient (4)
- My patients are satisfied with telehealth services (5)
- Telehealth allows me to offer services to patient who would not usually be able to attend my clinic (6)
- I find that telehealth is cost-effective for my practice (7)
- Telehealth is cost-effective for my patients (8)
- Telehealth offers a way of connecting with my clients, even if they are travelling (9)
- Telehealth reduces the number of patients who do not show up for appointments (10)
- Telehealth has reduced the need for physical space, saving on rent, utilities, and other overhead costs. (11)
- Telehealth allows me to offer appointments during hours that might not be feasible with in-person visits, such as early mornings or late evenings (12)
- Telehealth reduces the carbon footprint associated with patients traveling to and from the clinic (13)
- I can offer telehealth consultations from home, improving my work-life balance (14)
- Telehealth allows me to integrate digital health tools, apps, and wearables more easily into patient care (15)
- Telehealth facilitates easier collaboration with other healthcare professionals, allowing for more holistic patient care (16)
- Offering telehealth services gives my practice a competitive edge in the market (17)
- Other (18) __________________________________________________

Q44 In any given week since the easing of the COVID-19 pandemic restrictions, what proportion of your weekly patient caseload was provided as **individual videoconferencing consultations**:  *For example, if 70% of your caseload was provided as in-person, 15% was provided as individual consultations via videoconferencing, 10% was provided as group-based videoconferencing and 5% was provided as telephone, you should answer:* *15% for "individual videoconferencing consultations"*

|  | Not Applicable |
| --- | --- |

|  | 0 | 10 | 20 | 30 | 40 | 50 | 60 | 70 | 80 | 90 | 100 |
| --- | --- | --- | --- | --- | --- | --- | --- | --- | --- | --- | --- |

| Individual videoconferencing consultations () | 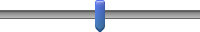 |
| --- | --- |

Q45 In any given week since the easing of the COVID-19 pandemic restrictions, what proportion of your weekly patient caseload was provided as **group-based videoconferencing consultations**:  *For example, if 70% of your caseload was provided as in-person, 15% was provided as individual consultations via videoconferencing, 10% was provided as group-based videoconferencing and 5% was provided as telephone, you should answer:* *10% for "group-based videoconferencing consultations"*

|  | Not Applicable |
| --- | --- |

|  | 0 | 10 | 20 | 30 | 40 | 50 | 60 | 70 | 80 | 90 | 100 |
| --- | --- | --- | --- | --- | --- | --- | --- | --- | --- | --- | --- |

| Group-based videoconferencing consultations () | 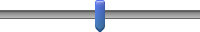 |
| --- | --- |

Q46 In any given week since the easing of the COVID-19 pandemic restrictions, what proportion of your weekly patient caseload was provided as **telephone consultations**:  *For example, if 70% of your caseload was provided as in-person, 15% was provided as individual consultations via videoconferencing, 10% was provided as group-based videoconferencing and 5% was provided as telephone, you should answer:* 5% for "telephone consultations".

|  | Not Applicable |
| --- | --- |

|  | 0 | 10 | 20 | 30 | 40 | 50 | 60 | 70 | 80 | 90 | 100 |
| --- | --- | --- | --- | --- | --- | --- | --- | --- | --- | --- | --- |

| Telephone consultations () | 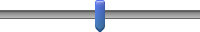 |
| --- | --- |

Q47 During the COVID-19 pandemic, what was your level of confidence providing physiotherapy consultations using telehealth?

- Not at all confident: 1 (1)
- 2 (2)
- 3 (3)
- 4 (4)
- 5 (5)
- 6 (6)
- 7 (7)
- 8 (8)
- 9 (9)
- Extremely confident: 10 (10)

Q48 During the COVID-19 pandemic, how effective did you find the physiotherapy care you provided to patients via telehealth?

- Not at all effective: 1 (1)
- 2 (2)
- 3 (3)
- 4 (4)
- 5 (5)
- 6 (6)
- 7 (7)
- 8 (8)
- 9 (9)
- Extremely effective: 10 (10)

Q49 During the COVID-19 pandemic, how satisfied were you with the physiotherapy care you provided to patients via telehealth?

- Not at all satisfied: 1 (1)
- 2 (2)
- 3 (3)
- 4 (4)
- 5 (5)
- 6 (6)
- 7 (7)
- 8 (8)
- 9 (9)
- Extremely satisfied: 10 (10)

| Page Break |  |
| --- | --- |

Q50 Thinking about the number of telehealth consultations you are providing now, is this less than what you intended offer as you emerged from the pandemic/restrictions?

- No, it's the same or more (1)
- Yes (2)

Display this question:

If Thinking about the number of telehealth consultations you are providing now, is this less than wh... = Yes

Q51 Why do you think you are offering telehealth less that you intended to (select all that apply)?

- Patient demand reduced more than expected after restrictions lifted (1)
- I do not like providing care via telehealth (2)
- The administrative burden of offering telehealth in addition to in-person consults is too high (3)
- My patients will not pay for telehealth (4)
- There is a lack of reimbursement by third party funders (5)
- I do not have access to suitable telehealth software/infrastructure (6)
- It is easier to do in-person consultations (7)
- I am not confident with offering telehealth (8)
- I am concerned with the safety of telehealth (9)
- I am concerned with the effectiveness of telehealth (10)
- The business cost of offering telehealth is too high (11)
- My patients prefer in-person services (12)

Display this question:

If Thinking about the number of telehealth consultations you are providing now, is this less than wh... = Yes

Q52 Are there any other reasons why you planned to not offer telehealth after the easing of the pandemic restrictions?

- No (1)
- Yes, please describe: (2) __________________________________________________

End of Block: After COVID Restrictions (from 2022)

Start of Block: Telehealth Information

Q53 What telehealth platform(s) have you most recently used (tick all that apply)?

- Zoom (1)
- Skype (2)
- Facetime (3)
- Physitrack (4)
- Coviu (5)
- Doxy.me (6)
- Vidyo (7)
- WhatsApp (8)
- Facebook Messenger (9)
- Microsoft Teams (10)
- Cliniko (11)
- Health Direct (12)
- eHAB (13)
- Telephone (14)
- Other, please specify: (15) __________________________________________________

Q79 Preferred fruit. If you are human, please select the last option

- Apple (1)
- Banana (2)
- Pear (3)
- Orange (4)
- Mango (5)

Q54 When consulting via telehealth, how often do you encounter technical issues?

- Never (1)
- Rarely (2)
- Sometimes (3)
- Often (4)
- Always (5)

Q55 When consulting via telehealth, how often do your patients encounter technical issues?

- Never (1)
- Rarely (2)
- Sometimes (3)
- Often (4)
- Always (5)

Q56 If you encounter technical issues, how disruptive are they to the consultation in general?

- Not at all (1)
- Slightly (2)
- Moderately (3)
- Very (4)
- Extremely (5)

Q57 When consulting with individual patients via telehealth, how often do you have to cancel/reschedule appointments due to technical issues?

- Never (1)
- Rarely (2)
- Sometimes (3)
- Often (4)
- Always (5)

Q58 When providing telehealth consultations to patients, do you use similar parameters of care to what you typically use in-person (i.e. similar consultation frequency, duration and similar content)?

- Yes (1)
- No, please explain the way in which your telehealth consultations differ. (2) __________________________________________________

Q59 Compared to your patients that you see in-person, how often do you see your patients via telehealth?

- Much more often (1)
- A little more often (2)
- About the same (3)
- A little less often (4)
- Much less often (5)

Q60 Compared to your patients that you see in-person, what is the duration of an average telehealth consultation:

- Much longer than an in-person consultation (1)
- A little longer than an in-person consultation (2)
- About the same as an in-person consultation (3)
- A little shorter than an in-person consultation (4)
- Much shorter than an in-person consultation (5)

Q61 Compared to your patients that you see in-person, the amount of money you charge for a telehealth consultation is:

- Much more than an in-person consultation (1)
- A little more than an in-person consultation (2)
- About / the same as an in-person consultation (3)
- A little less than an in-person consultation (4)
- Much less than an in-person consultation (5)

Q62 Thinking about your current weekly caseload, what proportion of patients do you offer a “hybrid” model of care to (i.e. some in-person consultations and some telehealth consultations)?

|  | 0 | 10 | 20 | 30 | 40 | 50 | 60 | 70 | 80 | 90 | 100 |
| --- | --- | --- | --- | --- | --- | --- | --- | --- | --- | --- | --- |

| Proportion of patients offered a hybrid model of care () | 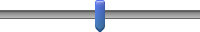 |
| --- | --- |

Display this question:

If Thinking about your current weekly caseload, what proportion of patients do you offer a “hybrid”... [ Proportion of patients offered a hybrid model of care ] > 0

Q63 If offering hybrid care, which of the following most applies to you and your patients?

- Patients typically receive many more in-person visits compared to telehealth visits (1)
- Patients typically receive some more in-person visits compared to telehealth visits (2)
- Patients typically receive about the same number of in-person and telehealth visits (3)
- Patients typically receive some less in-person visits compared to telehealth visits (4)
- Patients typically receive many less in-person visits compared to telehealth visits (5)

Q64 Are there any other details you would like to provide about how your model of care for telehealth consultations differs from a standard in-person consultation

- No (1)
- Yes, please describe: (2) __________________________________________________

| Page Break |  |
| --- | --- |

Q65 Since the easing of restrictions, how often have your patients requested telehealth consultations with you?

- Never (1)
- Rarely (2)
- Sometimes (3)
- Often (4)
- Frequently (5)

Q66 In your opinion, do patients like telehealth consultations:

- Much more than an in-person consultations (1)
- A little more than an in-person consultations (2)
- About / the same as an in-person consultations (3)
- A little less than an in-person consultations (4)
- Much less than an in-person consultations (5)

Q67 What is your experience with regard to the business costs of offering telehealth consultations versus in-person consultations:

- Telehealth consultations cost the business much more than in-person consultations (1)
- Telehealth consultations cost somewhat more than in-person consultations (2)
- Telehealth consultations and in-person consultations cost about the same (3)
- Telehealth consultations cost somewhat less than in-person consultations (4)
- Telehealth consultations cost the business much less than in-person consultations (5)

Q68 Please explain why you think that is:

________________________________________________________________

________________________________________________________________

________________________________________________________________

________________________________________________________________

________________________________________________________________

| Page Break |  |
| --- | --- |

Q69 Do you use any additional resources to support your videoconferencing consultations? (select all that apply)

- Text message reminders (1)
- Follow up phone calls (2)
- Apps for a smart phone or tablet (3)
- Wearables e.g. smart watch (4)
- Written/digital educational material about the issue/condition (5)
- Written/digital instructions, diagrams or booklets (6)
- Videos (7)
- Suggested websites for further information (8)
- Log books and diaries (9)
- Provision/purchase of equipment or devices (10)
- Other, please describe (11) __________________________________________________

Q70 Do you feel like delivering physiotherapy consultations via videoconferencing has become easier over time?

- No (1)
- Yes (2)

Q71 Since the easing of restrictions in 2022, what percentage of your patients have you felt were unsuitable for managing via telehealth?

|  | 0 | 10 | 20 | 30 | 40 | 50 | 60 | 70 | 80 | 90 | 100 |
| --- | --- | --- | --- | --- | --- | --- | --- | --- | --- | --- | --- |

| Percentage of patients unsuitable for telehealth () | 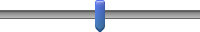 |
| --- | --- |

Q72 Of these, how often was the reason:

|  | Rarely/never (1) | Sometimes (2) | Often (3) |
| --- | --- | --- | --- |
| Patient complexity (1) |  |  |  |
| Complexity of problem/condition (2) |  |  |  |
| Patient did not have access to technology required or there were technical issues (3) |  |  |  |
| Patient unable to use technology (eg. blindness, cognitively impaired) (4) |  |  |  |
| Unable to adequately diagnose/assess patient (5) |  |  |  |
| Patient condition required hands-on treatment (6) |  |  |  |
| Safety concerns (7) |  |  |  |

Q73 Are there any other reasons that your clients have been unsuitable for telehealth?

- No (1)
- Yes, please describe: (2) __________________________________________________

Q74 Please add any further comments you have about providing telehealth consultations in clinical practice post-pandemic.

________________________________________________________________

________________________________________________________________

________________________________________________________________

________________________________________________________________

________________________________________________________________

Q75 Would you like to be entered into a draw to receive $1000 gift voucher in appreciation of completing this survey?

- No (1)
- Yes, please provide Name and Email address: (2) __________________________________________________

Q76 Thank you for taking the time to complete this survey. Do you consent to be contacted for future related research?

- No (1)
- Yes, please provide Name and Email address: (2) __________________________________________________

End of Block: Telehealth Information
