# Supplementary figures and images for: Telerehabilitation Trends in Australian Physiotherapy and an Exploration of Factors That Influence Use After COVID-19 Restrictions: Qualitative Content Analysis
Source: JMIR Rehabil Assist Technol. 2026 Jan 27;13:e81008. doi: 10.2196/81008 (PMC12844842; doi:10.2196/81008)

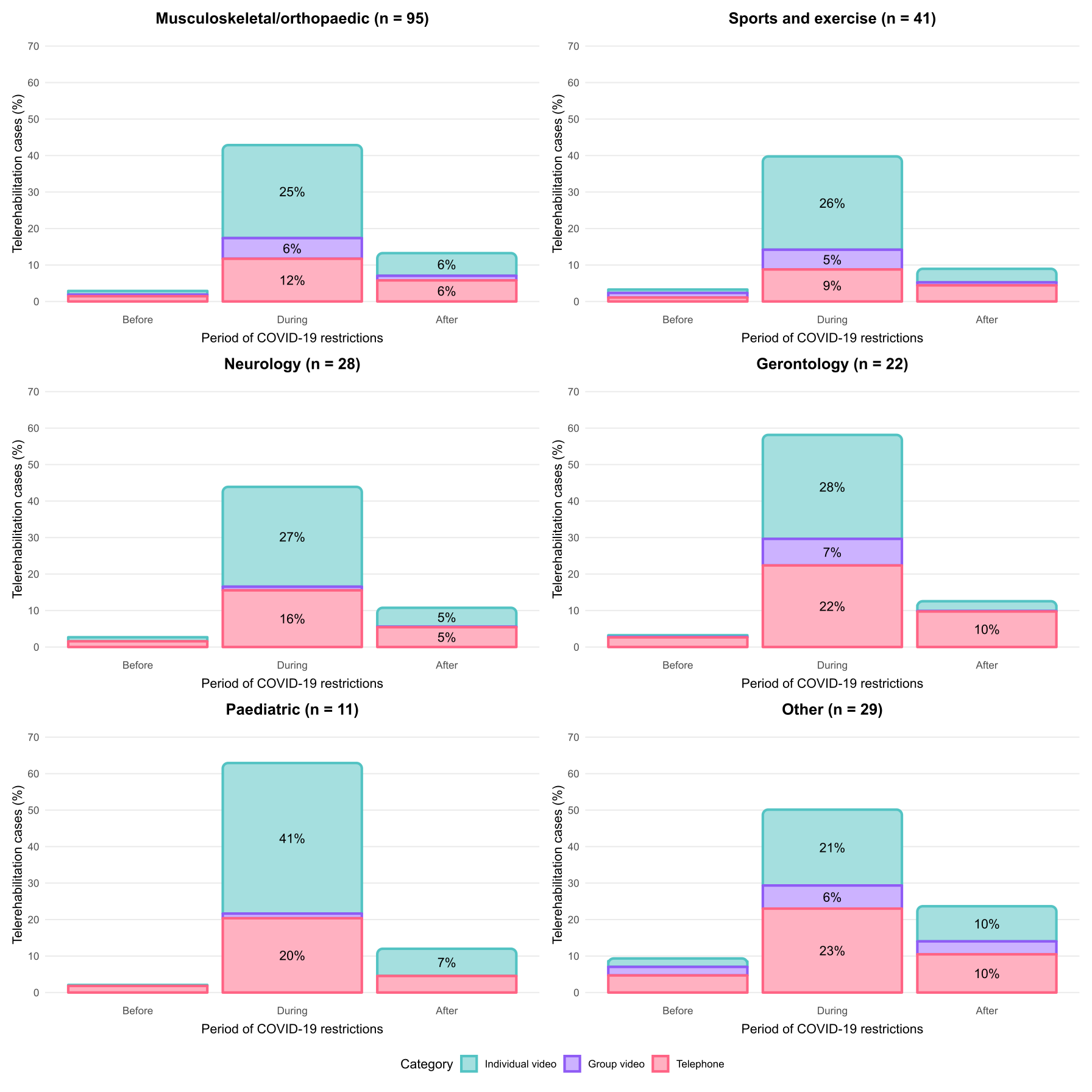

Supplement: Multimedia Appendix 2 [file rehab-v13-e81008-s002.png]

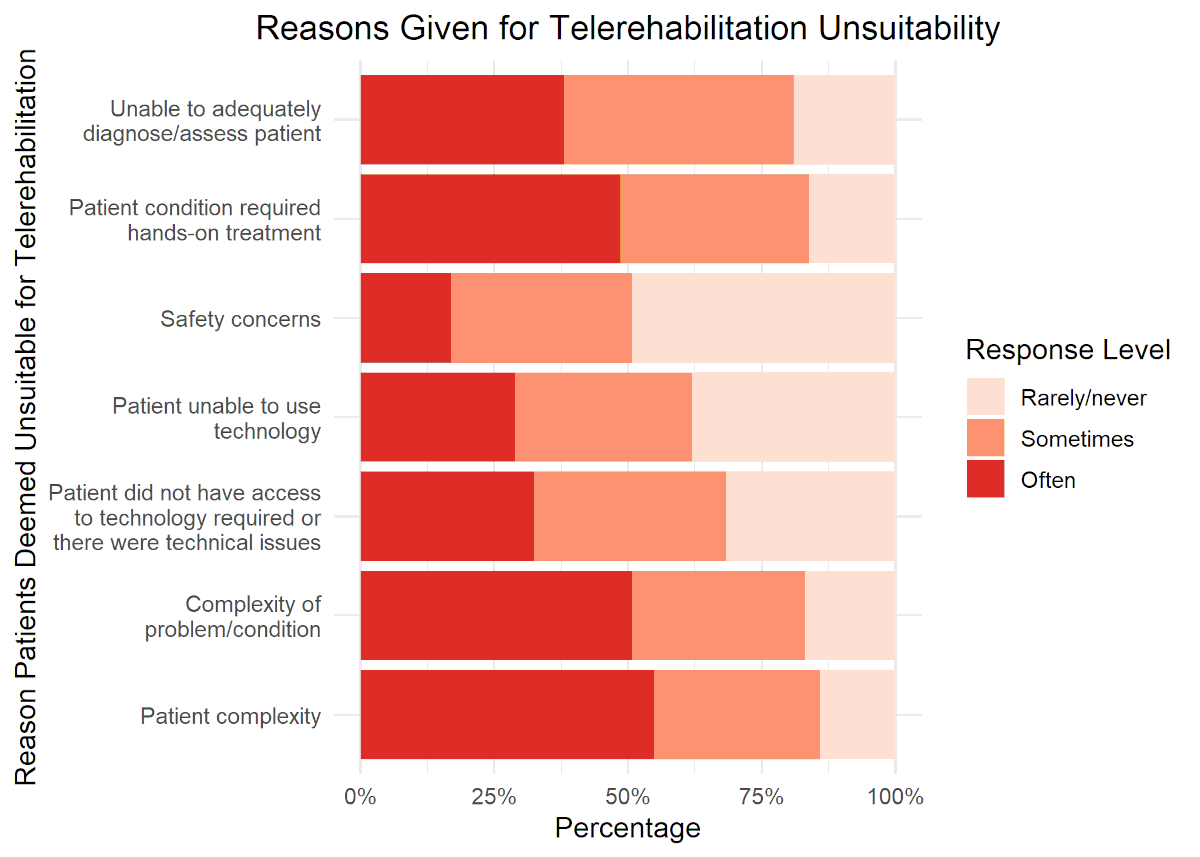

Supplement: Multimedia Appendix 4 [file rehab-v13-e81008-s004.png]

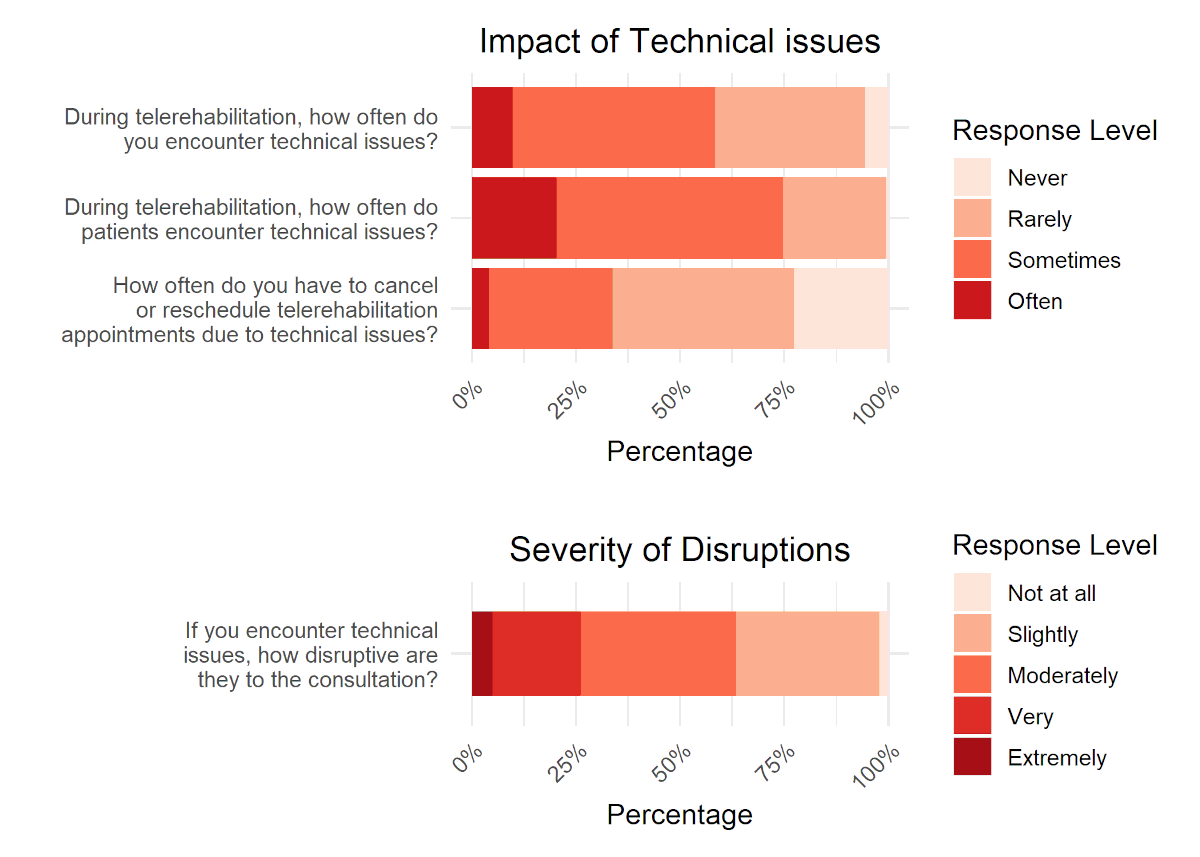

Supplement: Multimedia Appendix 5 [file rehab-v13-e81008-s005.png]
