# Supplementary material for: Telerehabilitation Trends in Australian Physiotherapy and an Exploration of Factors That Influence Use After COVID-19 Restrictions: Qualitative Content Analysis
Source: JMIR Rehabil Assist Technol. 2026 Jan 27;13:e81008. doi: 10.2196/81008 (PMC12844842; doi:10.2196/81008)
Supplement: Multimedia Appendix 3 [file rehab-v13-e81008-s003.docx]

Supplementary File 1

**Supplementary Table 1:** Reasons for not offering telerehabilitation prior to, during and after COVID-19 restrictions were lifted.

| **Reasons (n patients selecting choice)** | **Pre (n=104)** | | | **During (n=9)** | | **After (n=26)** | | |
| --- | --- | --- | --- | --- | --- | --- | --- | --- |
|  | **n** | **%** | **n** | | **%** | | **n** | **%** |
| Challenges with complex cases | 1 | 1 | - | | - | | - | - |
| Clients did not have access to suitable telehealth software/infrastructure and/or skills | 6 | 6 | - | | - | | - | - |
| I did not have access to suitable telehealth software/infrastructure | 47 | 45 | 1 | | 11 | | 1 | 4 |
| I didn’t see the need | 57 | 55 | 1 | | 11 | | 1 | 4 |
| I was concerned with the effectiveness of telehealth | 37 | 36 | 2 | | 22 | | 13 | 50 |
| I was concerned with the safety of telehealth | 10 | 10 | 0 | | 0 | | 6 | 23 |
| I was not aware of telehealth | 3 | 3 | - | | - | | - | - |
| I was not confident with offering telehealth | 29 | 28 | 2 | | 22 | | 3 | 12 |
| I was not practicing as a physiotherapist at the time | 3 | 3 | - | | - | | - | - |
| It was easier to do in-person practice | 55 | 53 | 2 | | 22 | | 20 | 77 |
| My patients preferred in-person services | 65 | 63 | 2 | | 22 | | 16 | 62 |
| My patients would not pay for telehealth | 24 | 23 | 1 | | 11 | | 5 | 19 |
| Telehealth was not supported by the organisation at the time | 14 | 13 | 1 | | 11 | | 2 | 8 |
| The business costs of offering telehealth was too high | 2 | 2 | 0 | | 0 | | 1 | 4 |
| There is a lack of reimbursement by third party funders | 22 | 21 | 0 | | 0 | | 2 | 8 |
| Were in the process of investigating feasibility of telehealth | 1 | 1 | - | | - | | - | - |
| I worked in a different area of physiotherapy during the pandemic | - | - | 2 | | 22 | | - | - |
| It was not appropriate for the setting I was working in | - | - | 1 | | 11 | | - | - |
| I did not like providing care via telehealth | - | - | - | | - | | 11 | 42 |
| Inertia from a long history of in-person care | - | - | - | | - | | 2 | 8 |
| My patients are not suitable for telehealth | - | - | - | | - | | 3 | 12 |
| The administrative burden of offering telehealth in addition to in-person consults is too high | - | - | - | | - | | 3 | 12 |

**Key:** “-” indicates this was not a response option provided to, or entered by, participants at that timepoint; “0” indicates it was a response option, but was not selected.

**Supplementary Table 2:** Reasons physiotherapists planned to stop offering telerehabilitation after restrictions eased (n=32)

| **Reason** | **n** | **%** |
| --- | --- | --- |
| It was easier to do in-person consultations | 23 | 72 |
| My patients prefer in-person services | 22 | 69 |
| I did not like providing care via telehealth | 18 | 56 |
| I was concerned with the effectiveness of telehealth | 17 | 53 |
| I was concerned with the safety of telehealth | 8 | 25 |
| I was not confident with offering telehealth | 7 | 22 |
| My patients would not pay for telehealth | 5 | 16 |
| There is a lack of reimbursement by third party funders | 4 | 13 |
| My practice decided to not offer telehealth consultations. | 2 | 6 |
| Clients did not have access to suitable telehealth software/infrastructure and/or skills | 2 | 6 |
| I did not have access to suitable telehealth software/infrastructure | 1 | 3 |
| The administrative burden of offering telehealth in addition to in-person consults is too high | 1 | 3 |
| The business cost of offering telehealth was too high | 1 | 3 |
| I didn't think it would be necessary to continue after restrictions were eased | 1 | 3 |
| Need for assistance on patient end | 1 | 3 |

**Supplementary Table 3:** Reasons physiotherapists were providing less telerehabilitation consultations than intended (n=58).

| **Reason** | **n** | **%** |
| --- | --- | --- |
| My patients prefer in-person services | 44 | 76 |
| Patient demand reduced more than expected after restrictions lifted | 32 | 55 |
| It is easier to do in-person consultations | 27 | 47 |
| I am concerned with the effectiveness of telehealth | 16 | 28 |
| I do not have access to suitable telehealth software/infrastructure | 8 | 14 |
| I do not like providing care via telehealth | 8 | 14 |
| My patients will not pay for telehealth | 7 | 12 |
| The administrative burden of offering telehealth in addition to in-person consults is too high | 7 | 12 |
| There is a lack of reimbursement by third party funders | 5 | 9 |
| I am concerned with the safety of telehealth | 3 | 5 |
| I am not confident with offering telehealth | 2 | 3 |
| The business cost of offering telehealth is too high | 2 | 3 |
| Clients did not have access to suitable telehealth software/infrastructure and/or skills | 1 | 2 |
| Building rapport and cultural safety are easier in person | 1 | 2 |
| Telehealth was not supported by the organisation at the time | 1 | 2 |

**Supplementary Table 4:** Reasons physiotherapists continued using telerehabilitation post-pandemic restrictions (n=118)

| **Reason** | **n** | **%** |
| --- | --- | --- |
| Telehealth allows me to offer services to patient who would not usually be able to attend my clinic | 84 | 71 |
| My patients like the option of receiving care via telehealth | 76 | 64 |
| My patients find telehealth convenient | 74 | 63 |
| Telehealth offers a way of connecting with my clients, even if they are travelling | 49 | 42 |
| I find that telehealth is effective | 40 | 34 |
| My patients are satisfied with telehealth services | 39 | 33 |
| I like providing care via telehealth | 29 | 25 |
| Telehealth reduces the number of patients who do not show up for appointments | 26 | 22 |
| Telehealth allows me to offer appointments during hours that might not be feasible with in-person visits, such as early mornings or late evenings | 17 | 14 |
| Telehealth is cost-effective for my patients | 17 | 14 |
| I can offer telehealth consultations from home, improving my work-life balance | 16 | 14 |
| I find that telehealth is cost-effective for my practice | 15 | 13 |
| Telehealth reduces the carbon footprint associated with patients traveling to and from the clinic | 13 | 11 |
| Telehealth allows me to integrate digital health tools, apps, and wearables more easily into patient care | 11 | 9 |
| Telehealth facilitates easier collaboration with other healthcare professionals, allowing for more holistic patient care | 11 | 9 |
| Telehealth has reduced the need for physical space, saving on rent, utilities, and other overhead costs. | 7 | 6 |
| Offering telehealth services gives my practice a competitive edge in the market | 5 | 4 |
| Telehealth allows continuity of care for patients who are fearful of or unable to access services due to their health condition | 2 | 2 |
| I am participating in a telehealth RCT | 1 | 1 |
| Telehealth reduces my travel for home visits | 1 | 1 |

**Supplementary Table 5:** Reasons for and frequency of patients being deemed unsuitable for telerehabilitation (n=142).

| **Question** | **Response** | **Count** | **%** |
| --- | --- | --- | --- |
| Patient complexity | Rarely/never | 20 | 14 |
|  | Sometimes | 44 | 31 |
|  | Often | 78 | 55 |
| Complexity of problem/condition | Rarely/never | 24 | 17 |
|  | Sometimes | 46 | 32 |
|  | Often | 72 | 51 |
| Patient did not have access to technology required or there were technical issues | Rarely/never | 45 | 32 |
|  | Sometimes | 51 | 36 |
|  | Often | 46 | 32 |
| Patient unable to use technology | Rarely/never | 54 | 38 |
|  | Sometimes | 47 | 33 |
|  | Often | 41 | 29 |
| Unable to adequately diagnose/assess patient | Rarely/never | 27 | 19 |
|  | Sometimes | 61 | 43 |
|  | Often | 54 | 38 |
| Patient condition required hands-on treatment | Rarely/never | 23 | 16 |
|  | Sometimes | 50 | 35 |
|  | Often | 69 | 49 |
| Safety concerns | Rarely/never | 70 | 49 |
|  | Sometimes | 48 | 34 |
|  | Often | 24 | 17 |

**Supplementary Table 6:** Additional reasons patients were deemed unsuitable for telerehabilitation (n=30).

|  | **n** | **(%)** |
| --- | --- | --- |
| Client preference is in-person | 6 | 20 |
| Physical examination is indicated | 4 | 13 |
| Complex patient presentations | 4 | 13 |
| Additional support is required at the client end | 3 | 10 |
| Hands-on treatment is indicated | 3 | 10 |
| When rapport has been difficult to build | 2 | 7 |
| Connectivity issues | 2 | 7 |
| Safety concerns | 1 | 3 |
| Multidisciplinary sessions | 1 | 3 |
| Social interaction would be beneficial | 1 | 3 |
| Additional time required for technology issues | 1 | 3 |
| Poor digital literacy of patient | 1 | 3 |
| Issues with payment/reimbursement | 1 | 3 |

**Supplementary Table 7:** Additional resources used to support videoconferencing consultations (n=142)

|  | **n** | **(%)** |
| --- | --- | --- |
| Text message reminders | 109 | 77 |
| Written/digital educational material about the issue/condition | 67 | 47 |
| Written/digital instructions, diagrams or booklets | 63 | 44 |
| Follow up phone calls | 54 | 38 |
| Suggested websites for further information | 53 | 37 |
| Videos | 49 | 35 |
| Apps for a smart phone or tablet | 45 | 32 |
| Provision/purchase of equipment or devices | 26 | 18 |
| Log books and diaries | 15 | 11 |
| Wearables e.g. smart watch | 11 | 8 |
| Email reminders | 1 | 1 |

**Supplementary Table 8:** How parameters of care (e.g., consultation frequency, duration and content) are different for telerehabilitation compared to in-person consultations (n=38).

| **Parameter** | **n** | **(%)** |
| --- | --- | --- |
| Limited physical assessment/treatment | 15 | 39 |
| Shorter consultations | 10 | 26 |
| More focused on exercise or education | 8 | 21 |
| More frequent consultations | 4 | 11 |
| Additional time for administration or technology issues | 3 | 8 |
| Less frequent consultations | 3 | 8 |
| Case management focus | 2 | 5 |
| As an adjunct to in-person consultations | 1 | 3 |
| Check in consultations | 1 | 3 |
| Difficult to interpret body language | 1 | 3 |
| Difficulties demonstrating | 1 | 3 |
| Funding model is different | 1 | 3 |
| Less confident in findings | 1 | 3 |
| Longer consultations | 1 | 3 |
| Only used when absolutely necessary | 1 | 3 |
| Parameters of care set by RCT | 1 | 3 |
| Unable to physically correct movement | 1 | 3 |

**Supplementary Table 9:** Parameters of care for telerehabilitation in clinical practice (n=142)

|  | **n** | **(%)** |
| --- | --- | --- |
| **Frequency of telerehabilitation consultations (vs in-person)** |  |  |
| Much less often | 23 | 16 |
| A little less often | 30 | 21 |
| About the same | 72 | 51 |
| A little more often | 13 | 9 |
| Much more often | 4 | 3 |
| **Duration of a telerehabilitation consultation (vs in-person)** |  |  |
| Much shorter than an in-person consultation | 14 | 10 |
| A little shorter than an in-person consultation | 53 | 37 |
| About the same as an in-person consultation | 61 | 43 |
| A little longer than an in-person consultation | 11 | 8 |
| Much longer than an in-person consultation | 3 | 2 |
| **Charge for a telerehabilitation consultation (vs in-person)** |  |  |
| Much less than an in-person consultation | 8 | 6 |
| A little less than an in-person consultation | 25 | 18 |
| About / the same as an in-person consultation | 104 | 73 |
| A little more than an in-person consultation | 4 | 3 |
| Much more than an in-person consultation | 1 | 1 |
| **Nature of hybrid telerehabilitation and in-person care (n=113)** |  |  |
| Patients typically receive many less in-person visits compared to telehealth visits | 6 | 5 |
| Patients typically receive some less in-person visits compared to telehealth visits | 15 | 13 |
| Patients typically receive about the same number of in-person and telehealth visits | 30 | 27 |
| Patients typically receive some more in-person visits compared to telehealth visits | 14 | 12 |
| Patients typically receive many more in-person visits compared to telehealth visits | 48 | 42 |
| **Frequency of patients requesting telerehabilitation after restrictions eased** |  |  |
| Never | 28 | 20 |
| Rarely | 69 | 49 |
| Sometimes | 42 | 30 |
| Frequently | 3 | 2 |
| **Physiotherapists’ perceive that patients like telerehabilitation** |  |  |
| Much less than an in-person consultations | 70 | 49 |
| A little less than an in-person consultations | 37 | 26 |
| About / the same as an in-person consultations | 30 | 21 |
| A little more than an in-person consultations | 4 | 3 |
| Much more than an in-person consultations | 1 | 1 |
| **Cost of telerehabilitation to the business** |  |  |
| Much less than an in-person consultation | 17 | 12 |
| Somewhat less than an in-person consultation | 34 | 24 |
| About the same as an in-person consultation | 71 | 50 |
| Somewhat more than an in-person consultation | 13 | 9 |
| Much more than an in-person consultation | 7 | 5 |

**Supplementary Table 10:** Ways in which telerehabilitation models of care differ from in-person models (n=27)

|  | **n** | **(%)** |
| --- | --- | --- |
| Used in a hybrid model after initial in-person consult | 8 | 30 |
| Limited physical assessment/treatment | 4 | 15 |
| Case management focus | 3 | 11 |
| Lower uptake and attendance | 2 | 7 |
| Provides greater access to care | 2 | 7 |
| Allocate additional time for administration and technology issues | 1 | 4 |
| Benefits of all telehealth model rather than hybrid | 1 | 4 |
| Greater data and privacy concerns | 1 | 4 |
| More difficult to build rapport | 1 | 4 |
| Not suitable for all patients | 1 | 4 |
| Used as an adjunct or alternative when in-person is not possible | 1 | 4 |

**Supplementary Table 11:** Perspectives about providing care via telerehabilitation in clinical practice post-pandemic (qualitative content analysis)

| **Theme / Code** | **Frequency** |
| --- | --- |
| **Concerns** | **28** |
| Clients prefer/seek in person consultations | 12 |
| Not suitable for all clients | 4 |
| Concerns about assessment | 2 |
| Infrastructure (internet) can be an issue | 2 |
| issues with payment/reimbursement | 2 |
| Easier to build rapport in-person | 2 |
| Issues getting referrals for telerehabilitation | 1 |
| Software issues | 1 |
| Staff:patient ratios higher for telerehabilitation | 1 |
| Outcomes/attendance rates poorer in telerehabilitation | 1 |
| **How it is used** | **8** |
| Used in subsequent consultations | 2 |
| Videoconsultations are better than telephone consultations | 2 |
| For exercise prescription | 1 |
| Telerehabilitation for triaging purposes | 1 |
| Used for case management | 1 |
| Require support at the patient end | 1 |
| **Benefits** | **20** |
| Improves patient access | 8 |
| Increased acceptance of telerehabilitation | 6 |
| Telerehabilitation is effective in specific conditions/presentations | 3 |
| Encourages self-management | 2 |
| Provides greater flexibility | 1 |
| **Physiotherapists willingness to provide telerehabilitation services** | **23** |
| Willing to provide telerehabilitation services | 16 |
| Physiotherapist satisfaction with telerehabilitation is low | 2 |
| Physiotherapists prefer in-person consults | 2 |
| Physiotherapist education about how to deliver telerehabilitation is better | 1 |
| Participating in a telehealth RCT | 1 |
| Education about utility of telerehabilitation for physiotherapy is needed | 1 |
